# Red and White Chinook Salmon (*Oncorhynchus tshawytscha*): Differences in the Transcriptome Profile of Muscle, Liver, and Pylorus

**DOI:** 10.1007/s10126-020-09980-5

**Published:** 2020-06-26

**Authors:** Angelico Madaro, Ole Torrissen, Paul Whatmore, Santosh P. Lall, Jerome Schmeisser, Viviane Verlhac Trichet, Rolf Erik Olsen

**Affiliations:** 1 Institute of Marine Research, Animal Welfare Science Group, 5984 Matredal, Norway; 2 National Research Council of Canada, Institute for Marine Biosciences, Halifax, NS B3H 3Z1 Canada; 3 Research Centre of Animal Nutrition and Health–DSM Nutritional Products France, BP 170, 68305 Saint-Louis CEDEX, France; 4 Department of Biology, Norwegian University of Science and Technology, 7491 Trondheim, Norway

**Keywords:** *Oncorhynchus tshawytscha*, Red/white Chinook, Transcriptome analyses, Midgut–hindgut muscle, Astaxanthin, Pigmentation

## Abstract

Astaxanthin (Ax), the main carotenoid responsible for the distinct red flesh color in salmonids (Oncorhynchus, Salvelinus, Salmo, and Parahucho), is added to the diet of farmed fish at a substantial cost. Despite the great economical value for the salmon industry, the key molecular mechanisms involved in the regulation of muscle coloration are poorly understood. Chinook salmon (*Oncorhynchus tshawytscha*) represent an ideal model to study flesh coloration because they exhibit a distinct color polymorphism responsible for two color morphs, white and red flesh pigmented fish. This study was designed to identify the molecular basis for the development of red and white coloration of fish reared under the same experimental conditions and to better understand the absorption mechanism of Ax in salmonids. Pyloric caeca, liver, and muscle of both groups (*n* = 6 each) were selected as the most likely critical target organs to be involved respectively in the intestinal uptake, metabolism, and retention of Ax. Difference in the transcriptome profile of each tissue using next-generation sequencing technology was conducted. Ten KEGG pathways were significantly enriched for differentially expressed genes between red and white salmon pylorus tissue, while none for the transcriptome profile in the other two tissues. Differential expressed gene (DE) analyses showed that there were relatively few differences in muscle (31 DE genes, *p* < 0.05) and liver (43 DE genes, *p* < 0.05) of white and red Chinook salmon compared approximately 1125 DE genes characterized in the pylorus tissue, with several linked to Ax binding ability, absorption, and metabolism.

**Electronic supplementary material:**

The online version of this article (10.1007/s10126-020-09980-5) contains supplementary material, which is available to authorized users.

## Introduction

Carotenoids are responsible for the bright yellow to red color in terrestrial and aquatic animals. Most vertebrates cannot synthesize carotenoids de novo and must obtain them from their diets. Astaxanthin (Ax, 3,3′-dihydroxy-β,β-carotene-4,4′-dione) is the major carotenoid in wild salmonids (Schiedt et al. 1988; Torrissen 1989) and originates mainly from ingested zooplankton. Among salmonid fishes, four genera (*Oncorhynchus*, *Salmo*, *Salvelinus*, *Parahucho*) show distinct pigmented flesh, skin, and eggs. Mainly astaxanthin and to some extend canthaxanthin (Cx, β,β-carotene-4,4′-dione), are the two primary carotenoid pigments widely used to impart unique pinkish-red color to flesh of farmed salmonids. Several important biotic and abiotic factors can also influence the degree and retention of carotenoids in salmonid fish. Ax retention in fish flesh is particularly affected by the efficiency of absorption from the digestive tract, transport by lipoproteins, biochemical mechanisms involved in tissue uptake, and excretion and genetic factors, which have been the subject of several reviews (Rajasingh et al. 2007; Amaya and Nickell 2015; Baranski 2015; Lim et al. 2018). Liver, kidney, and gastrointestinal tract appear to play significant roles in carotenoids biotransformation and elimination, with the liver appearing to play the largest role (Schiedt et al. 1986). Ax is present predominantly in muscle in the free form whereas as esters in the skin. In muscle, Ax is loosely bound to α-actinin, and the ability to bind to protein does not show any difference between Atlantic salmon and white-fleshed fish (halibut and haddock) (Saha et al. 2005; Matthews et al. 2006).

Although significant progress has been made to better understand the biochemical mechanisms involved in regulating the absorption, transport, tissue uptake, and metabolism of carotenoids particularly in Atlantic salmon, many questions remain unanswered. After absorption, lipoproteins transport Ax to the liver and other tissues. Among the lipoproteins, the highest concentrations of Ax and Cx have been found in HDL or high-density lipid fraction (Aas et al. 1999; Chimsung et al. 2013). The distribution of Ax in various lipoproteins can be influenced by dietary cholesterol levels (Chimsung et al. 2013). Carotenoids are hydrophobic in nature and thus require class B scavenger proteins (Scarb) to transport them into the cell (Kiefer et al. 2002). In Atlantic salmon, *scarb1* is highly expressed in midgut as compared with other tissues (liver and skeletal muscle) (Sundvold et al. 2011).

Two carotenoid cleavage enzymes have been identified in birds, mammals and fish, BCO1 and BCO2. BCO1 promotes the symmetric cleavage of provitamin A carotenoids such as β- and α-carotene to produce two molecules of retinal (Olson and Hayaishi 1965), whereas BCO2 is responsible for the asymmetric cleavage of carotenes such as β-carotene and also xanthophyll carotenoids such as lutein and zeaxanthin (Mein et al. 2011). However, latest studies (Zoric 2017; Helgeland et al. 2019) showed that two paralog of the same gene in salmon *bco1* and *bco1*-*like* may act differently, with *bco1*-*like* being an active carotenoid oxygenase, with 15,15′- oxygenase activity. Although, in the same assay, *bco1* did not show any cleavage activity. Additional studies are needed to further examine the role of these two enzymes in carotenoid metabolism.

Recently, two molecular studies in coloration of two mutant bird systems: the yellowbeak in zebra finch mutant (Mundy et al. 2016) and the red factor in canary (Lopes et al. 2016) have identified a gene, *Cyp2j19*, as a ketolase responsible for the conversion of yellow dietary carotenoids into red ketocarotenoids. A single copy of *Cyp2j19* appears to be widespread across avian lineages (Twyman et al. 2018). *Cyp2j19* is a member of the Cytochrome P450 family of monooxygenases, which has diverse roles in a range of cellular systems, including detoxification.

Chinook salmon (*Oncorhynchus tshawytscha*) represent an ideal model to study Ax metabolism because they exhibit a distinct color polymorphism, resulting in two color morphs, white and red, with some individuals that do not deposit Ax in muscle, eggs, and skin (Rajasingh et al. 2007; Baranski 2015; Lehnert et al. 2019). Interestingly, these genetic polymorphisms are highly heritable (Withler 1986). Recently, Lehnert (2016) identified 90 single nucleotide polymorphisms (SNPs) associated with carotenoid pigmentation. Several genes throughout the genome were responsible for carotenoid coloration in Chinook salmon. The present study was designed to identify the molecular basis for the development of red and white Chinook salmon. As the organ or tissue governing the pigmentation difference is yet unknown, we focused on three likely target organs, pyloric caeca, liver, and muscle that regulate intestinal uptake, metabolism, and retention, respectively. Six biological replicates for tissue were selected for both white and red Chinook salmon and subjected to transcriptome analysis performed by mRNA sequencing technology.

## Materials and Methods

### Ethics Statement

This work was conducted in accordance with the laws and regulations controlling experiments and procedures on live animals in Norway.

### Experimental Animals and Facilities

Fertilized white and red Chinook eggs were obtained from Little Port Walter Facility (Alaska) and transported to the Institute of Marine Research in Matre (Norway), where they were hatched and reared. When juveniles had reached the parr stage, they were transferred into six squared 1.5-m (volume 1200 L) tanks filled with freshwater of 9.4 °C (± 0.5) and randomly divided into groups of around of 100 individuals per tank. Tanks were covered with a lid furnished with two neon tubes and a 24-h light regime was applied. At the average size of 60 g, Chinook salmon were induced to smoltify by light-controlled system (6 weeks 12 h L:12 h D followed by 6 weeks 24L:0D, 9 °C). Both red and white Chinook were fed ad libitum with pellet supplemented with 100 mg/kg astaxanthin (Nutra Olympic 5 mm, Skretting, Norway), which were delivered continuously throughout the 24-h cycle by automatic feeders (Arvo-Tec T drum 2000).

**Fig. 1:**
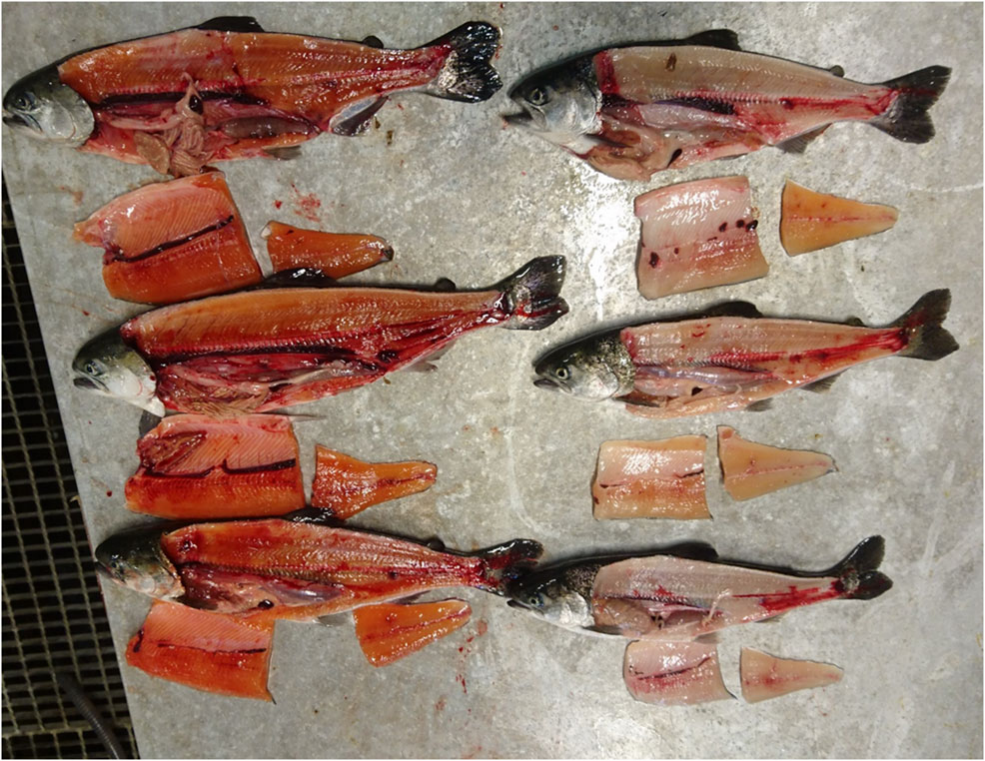
A sample of the Chinook salmon white and red individuals selected for this study

On the day of the sampling, 6 white Chinook salmon of about 259.6 ± 56 g weight and 26.2 ± 1.7 cm of length and 6 red Chinook salmon of 226.5 ± 82 g weight and 25.3 ± 3,4 cm of length were visually selected according the muscle pigmentation (Fig. 1) and sacrificed with an overdose of anesthesia (100 mg L − 1, Finquel®vet., ScanAqua AS, Årnes, Norway). Samples of muscle, liver, and pylorus were collected for each fish and stored in RNAlater (RNA*later*® RNA Stabilization Solution, Life Technology, Oslo, Norway) at 4 °C overnight and subsequently at − 80 °C until isolation of total RNA.

### RNA Extraction

RNA extraction was carried out at DSM Nutritional Products, France. Muscle liver and pylorus total RNA were isolated using RNeasy Plus Universal Mini Kit (Qiagen, Hilden Germany) according to the manufacturer’s instructions. RNA concentration and purity were determined using a NanoDrop ND-1000 spectrophotometer (Thermo Fisher Scientific) and by Qubit® 2.0 fluorometer RNA quantification (Thermo Fisher Scientific). Furthermore, RNA integrity was assessed by using Agilent 2100 Bioanalyzer (Agilent Technologies, Santa Clara, CA, USA). All samples had an RNA Integrity Number (RIN) equal or above eight. Six samples from red Chinook and six samples for white Chinook salmon were selected to construct the sequencing libraries.

### Library Preparation and Sequencing

A total of 36 samples—6 white and 6 red Chinook salmons, × 3 tissue types (liver, muscle and pylorus)—were sent for library preparation and sequencing by Helixo Genomics service provider in France. NEBNext® (New England Biolabs) library preparation, magnetic isolation, and multiplexing kits for Illumina were used with 500 ng of total RNA per sample. Target mRNA was first fragmented and bound to random primers. Amplification of cDNA from these fragments was performed using a reverse transcriptase lacking in RNase H activity to minimize RNA degradation, with actinomycin D added during first strand CDNA synthesis to inhibit DNA polymerase activity and dUTP added during second strand synthesis to label second strands. Double stranded cDNA fragments were then separated from the second strand reaction mix using AMPure XP beads. To prevent fragments from ligating to each other, a single adenine was added to the 3′ fragment ends and a following single thymine was also added to reduce chimera formation. The second strand was then removed with the USER enzyme and the remaining single stranded cDNA extracted using AMPure XP beads. Finally, the ss cDNA was PCR amplified over 11 cycles and the PCR products removed with AMPure XP beads. During the PCR amplification step, Illumina adapters and a multiplexing barcode was attached to the amplicon 3′ ends. After verifying RNA amplicon quality and size on a Bioanalyzer, samples were normalized to 10 nM and pooled into 3 total pools, with 12 samples per pool (where each sample per pool contained a different barcode). Pooled libraries were denatured with 0.2 N NaOH and diluted to 20 pM. Single read sequencing was completed on a NextSeq 500 using a NextSeq 500 High Output v2 kit.

### Sequence Quality Control, Genome Alignment, and Expression Quantification

Sequence reads were converted from BCL to fastq file format, quality checked, filtered, demultiplexed, and had PCR adapters removed using Bcl2fastq 2.0 (Illumina) software. Reads with a quality score of less than Q30 and a barcode mismatch of 1 base or more were removed.

When analysis of the data for this project commenced, no Chinook salmon reference genome was available. Because of this, reads were initially mapped according to reference genomes of three related species: Coho salmon (Okis_V1), rainbow trout (Omyk_1.0), and Atlantic salmon (ICSASG_v2). Recently, the Chinook salmon genome was published (Otsh_V1.0), and so reads were remapped to the Chinook genome. Downstream analysis (e.g. differential expression and functional annotation) is based on these Chinook mapping results. There were some interesting associations in the mapping results between the four species; thus, mapping results for the three non-Chinook species have been included in the initial results section.

The genomes were first indexed, and then reads were aligned to the indexed genomes using the default parameters in HISAT2 v2.1.0 (Kim et al. 2015). From the Chinook mapping results, total mRNA expression per gene was quantified using the default parameters of featureCounts v1.6.0 (Liao et al. 2014) (Liao et al. 2014) and genome feature definitions from the reference genome General Feature Format (GFF) annotation. This produced a count table of sequence reads per gene that was used in downstream differential expression analysis.

Fastq files containing quality filtered reads have been uploaded to the National Center for Biotechnology Information (NCBI) Sequence Read Archive (SRA). Project accession number is PRJNA591068.

### Differential Expression Analysis and Metabolic Pathway Enrichment

Downstream analysis of read counts per gene, including examination of genes that were differentially expressed (DE) between white and red salmon, was completed in R v3.4.4 (http://cran.rproject.org/). Data structure was examined and validated prior to DE analysis, which included outliers and batch effect assessment, using base R tools to produce PCA plots, read density plots, hierarchical clustering, and pairwise sample comparisons. The DESeq2 package (Love et al. 2014) was used for DE analysis based on 3 experimental group comparisons: white salmon livers vs red salmon livers, white salmon muscle vs red salmon muscle, and white salmon pylorus vs red salmon pylorus. DESeq2 fits counts per gene to a negative binomial generalized linear model (GLM), estimating log2 fold change and expression strength of each gene between experimental groups. A Wald test is used to test the significance of gene expression and the *p* values are false discovery rate (FDR) tested using the Benjamini-Hochberg method (Benjamini and Hochberg 1995). Genes with an FDR adjusted *p* value of less than 0.05 were considered to be significant.

Functional enrichment in KEGG (Kyoto Encyclopedia of Genes and Genomes) pathways and GO (Gene Ontology) terms was assessed based on the list of significantly DE genes using the ClusterProfiler package v3.6.0 (Yu et al. 2012). ClusterProfiler is an analysis and visualization module that is based on statistical analysis from the DOSE (Disease Ontology Semantic and Enrichment) package (Yu et al. 2015). DOSE performs a hypergeometric test to estimate overrepresentation of DE genes per pathway. As with DE analysis, significance values were FDR tested using Benjamini-Hochberg. KEGG pathways and GO terms were significantly enriched if they had FDR adjusted *p* values of less than 0.05.

## Results

### Sequencing, Transcript Identification, and Annotation

A total of 1,628,701,408 single-end, 75 bp reads were sequenced. On average, 92.56% of reads per sample passed quality filters (≥ Q30 and no barcode mismatches), resulting in 1418 million “clean” reads and an average of 38.67 million reads per sample.

As discussed in the method selection, reads were mapped to four salmonid species: Chinook salmon, Coho salmon, rainbow trout, and Atlantic salmon, through downstream results are based only on Chinook salmon mapping results. As expected, mapping against the recently published Chinook salmon genome produced the best results—around 85%–90% total mapping rate (Supplementary table 1 and 2). Interestingly, the mapping rate for uniquely mapped reads was very similar between Chinook and Coho salmon, indicating their close relatedness. However, the greater overall mapping rate for the Chinook genome was due to considerably higher multimapping than found in the Coho salmon. This further supports the “whole genome duplication and divergence” evidence for salmonids, showing that though the Chinook salmon genome is very similar to the Coho salmon, the main difference is the variability of isoforms between the two. Overall, the mapping rate was still relatively high for Coho salmon and rainbow trout genomes, around 70%–85% against the Coho salmon genome and 60%–80% against the rainbow trout genome and was reasonable for Atlantic salmon (about 40%–60%).

### Within and Between-Group Variance

**Fig. 2:**
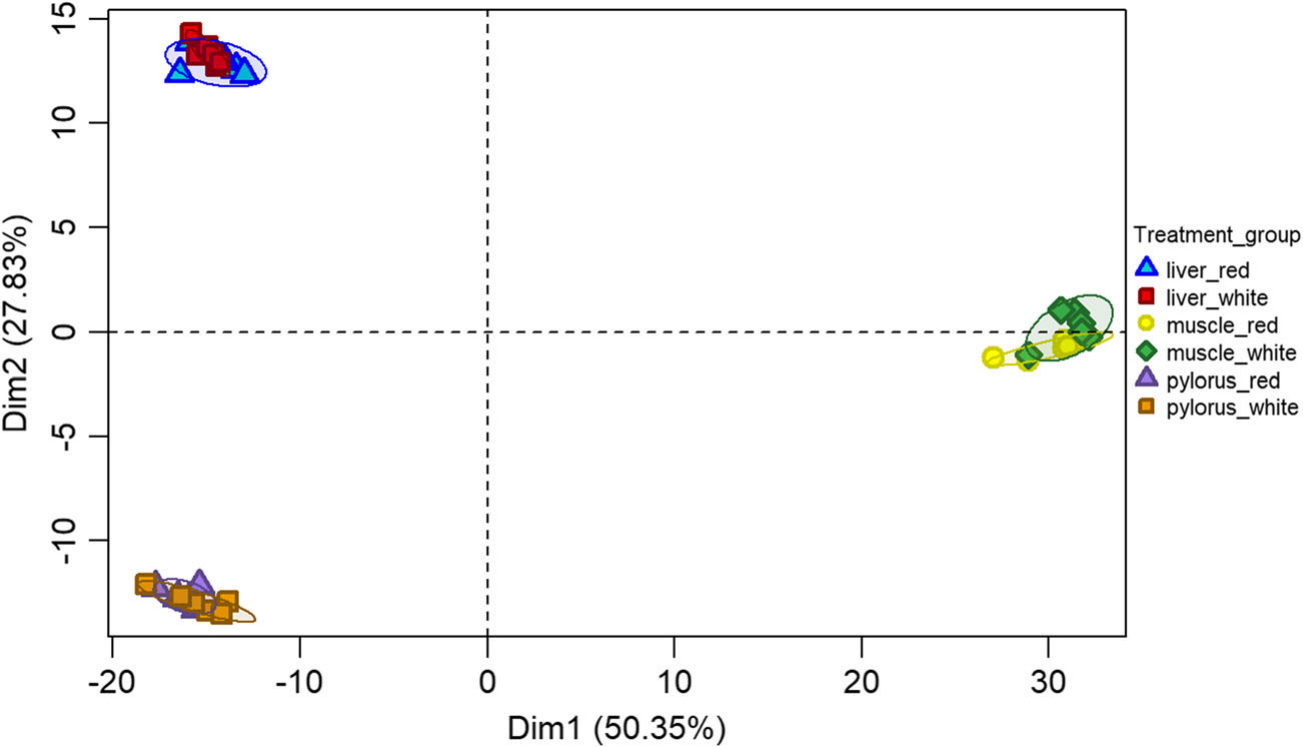
Principal component analysis (PCA) performed on the transcription data of muscle, liver, and pylorus of both red and white Chinook salmon. Groups have been colored and shaded using the R package “vegan” and are based on 85% confidence levels for data range

**Fig. 3:**
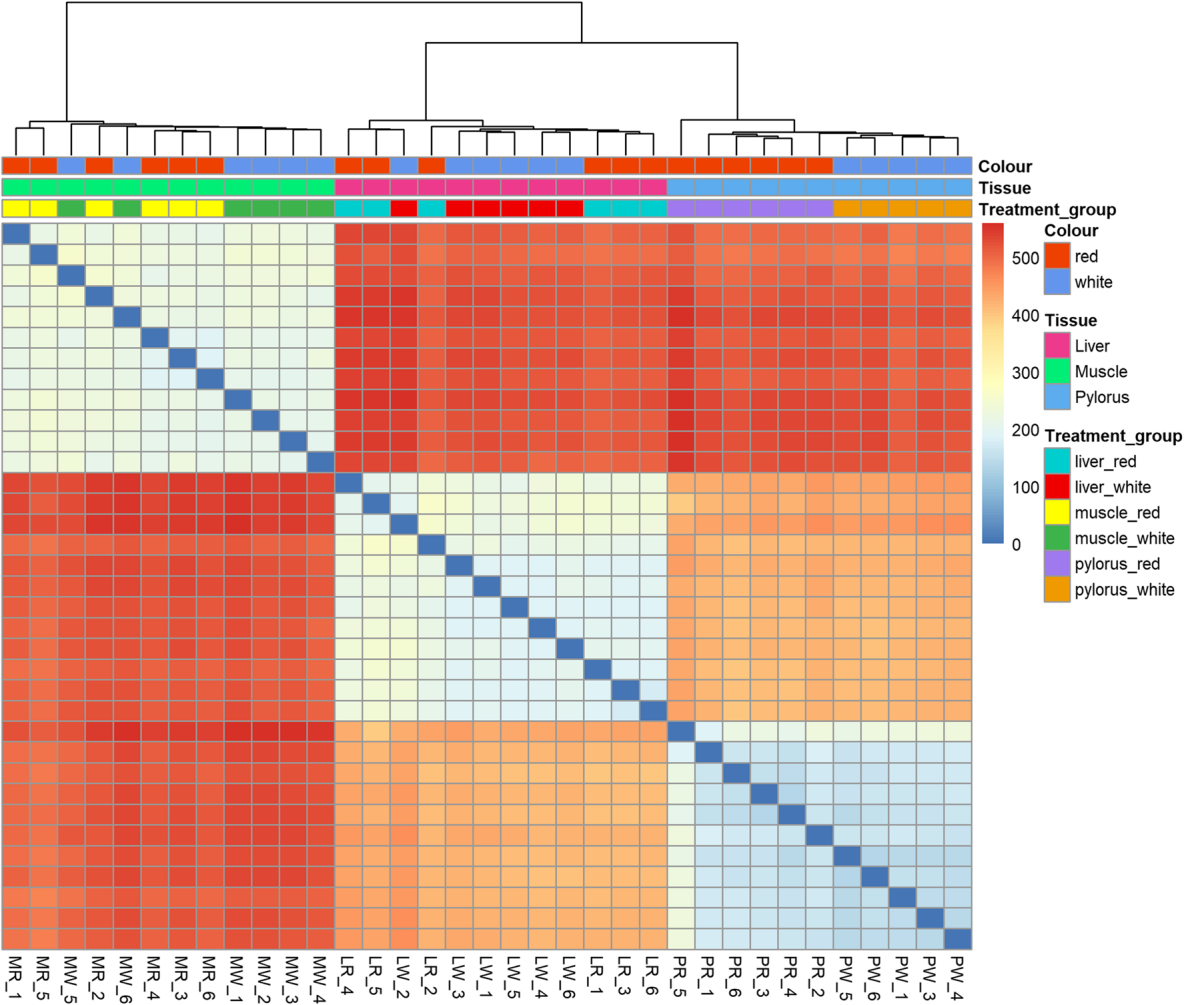
Pairwise distance clustering of red and white Chinook salmon samples (dendrogram and heatmap). Distance scale (0:500) indicates Euclidean distance between samples as calculated by the base R package “dist” (http://cran.rproject.org/)

Individual samples clustered primarily, and strongly, according to tissue type, i.e. liver, muscle, and pylorus (Fig. 2; for single tissue PCA plots: Supplementary figure 1A, 1B, and 1C). In the pylorus, red and white Chinook samples clustered separately, and there was some but not complete separation between red and white Chinook in muscle and liver samples (Heatmap—Fig. 3).

### Differentially Expressed Genes

**Fig. 4:**
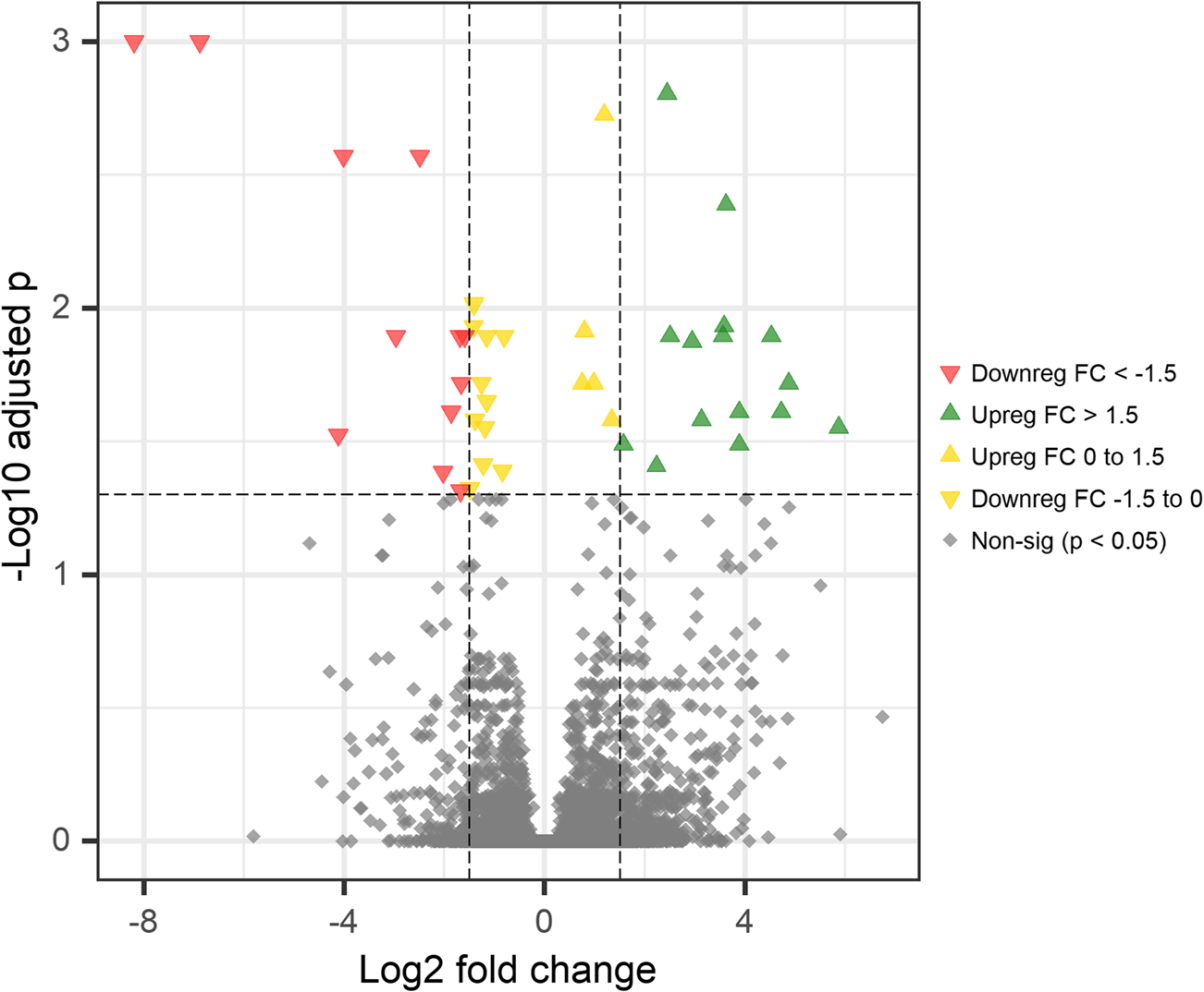
Volcano plot showing upregulated and downregulated genes comparing white and red Chinook salmon (*Oncorhynchus tshawytscha*) liver. The y-axis represents Benjamini-Hochberg adjusted *p* value, and the x-axis represents the log2 fold change of mean read counts per gene between red and white Chinook salmon livers

**Fig. 5:**
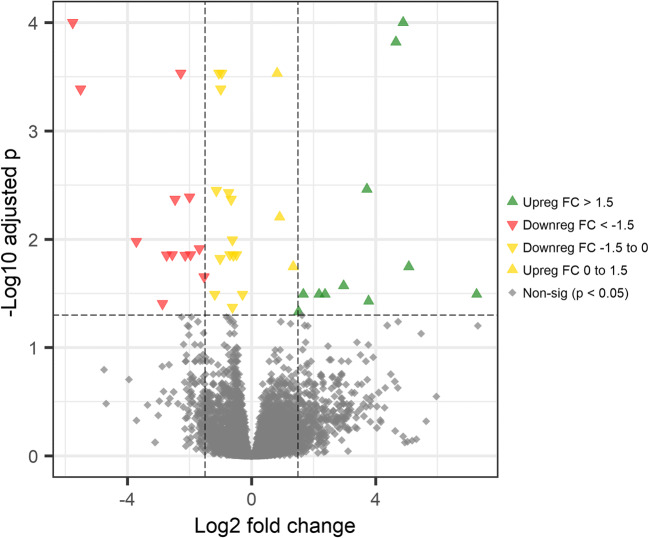
Volcano plot showing upregulated and downregulated genes comparing white and red Chinook salmon (*Oncorhynchus tshawytscha*) muscle. The y-axis represents Benjamini-Hochberg adjusted *p* value, and the x-axis represents the log2 fold change of mean read counts per gene between red and white Chinook muscle

**Fig. 6:**
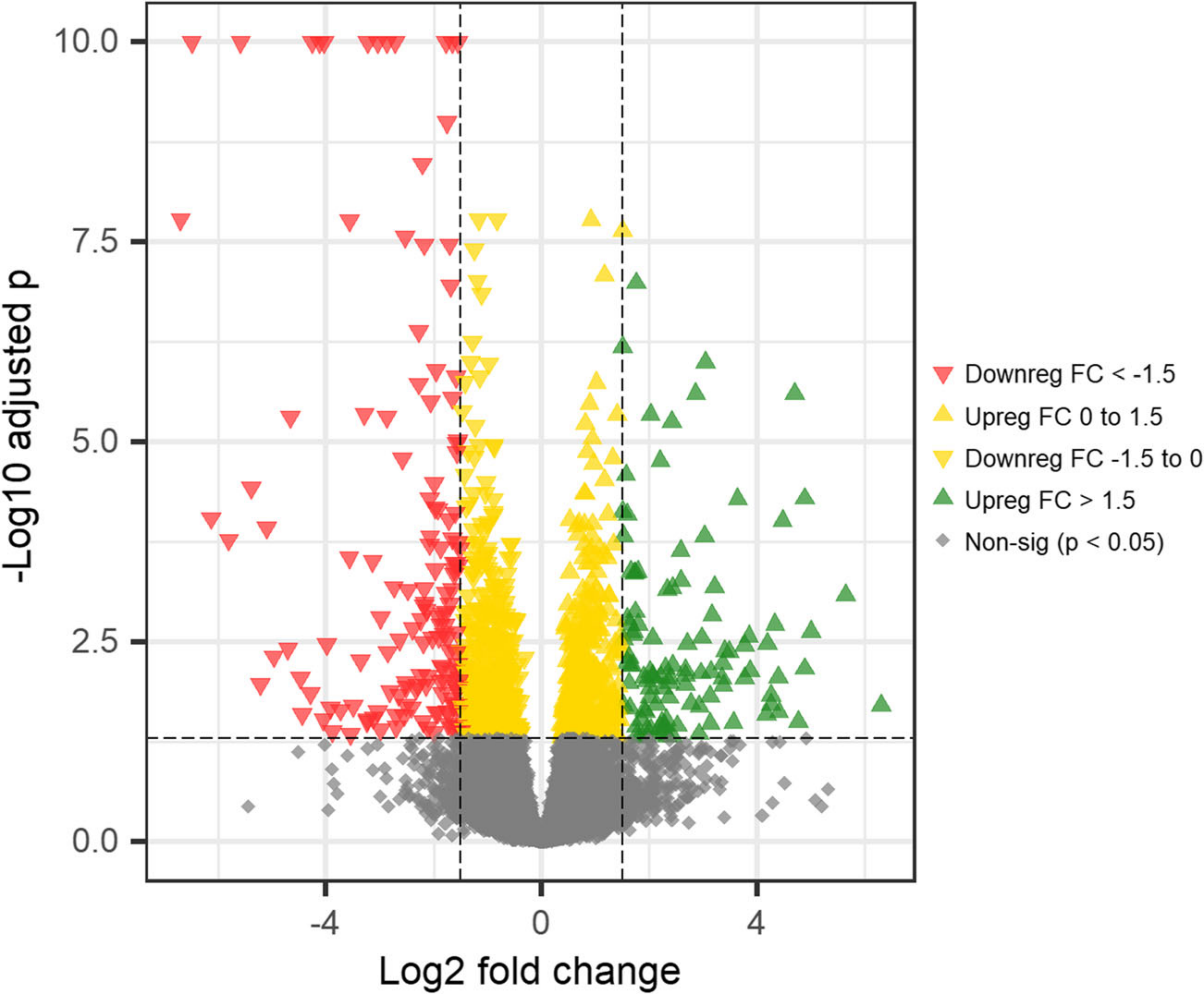
Volcano plot showing upregulated and downregulated genes comparing white and red Chinook salmon (*Oncorhynchus tshawytscha*) pylorus. The y-axis represents Benjamini-Hochberg adjusted *p* value, and the x-axis represents the log2 fold change of mean read counts per gene between red and white Chinook salmon pylorus

In the liver, there were 43 significantly (FDR adjusted *p* < 0.05) differentially expressed genes between red and white Chinook groups, with 20 genes upregulated (log2 fold change > 0) and 23 genes downregulated (log2 fold change < 0; Fig. 4), and in muscle, there were 31 differentially expressed genes with 14 upregulated and 27 downregulated (Fig. 5). The pyloric caeca had the highest level of transcriptome expression differences between red and white Chinook groups, with 1125 differentially expressed genes detected, of which 555 were upregulated and 570 downregulated (Fig. 6).

The entire list of differentially expressed genes is provided as supplementary data for liver (Supplementary Table 3) muscle (Supplementary file 4), and pylorus (Supplementary file 5), respectively.

### KEGG Pathways and GO Terms

**Fig. 7:**
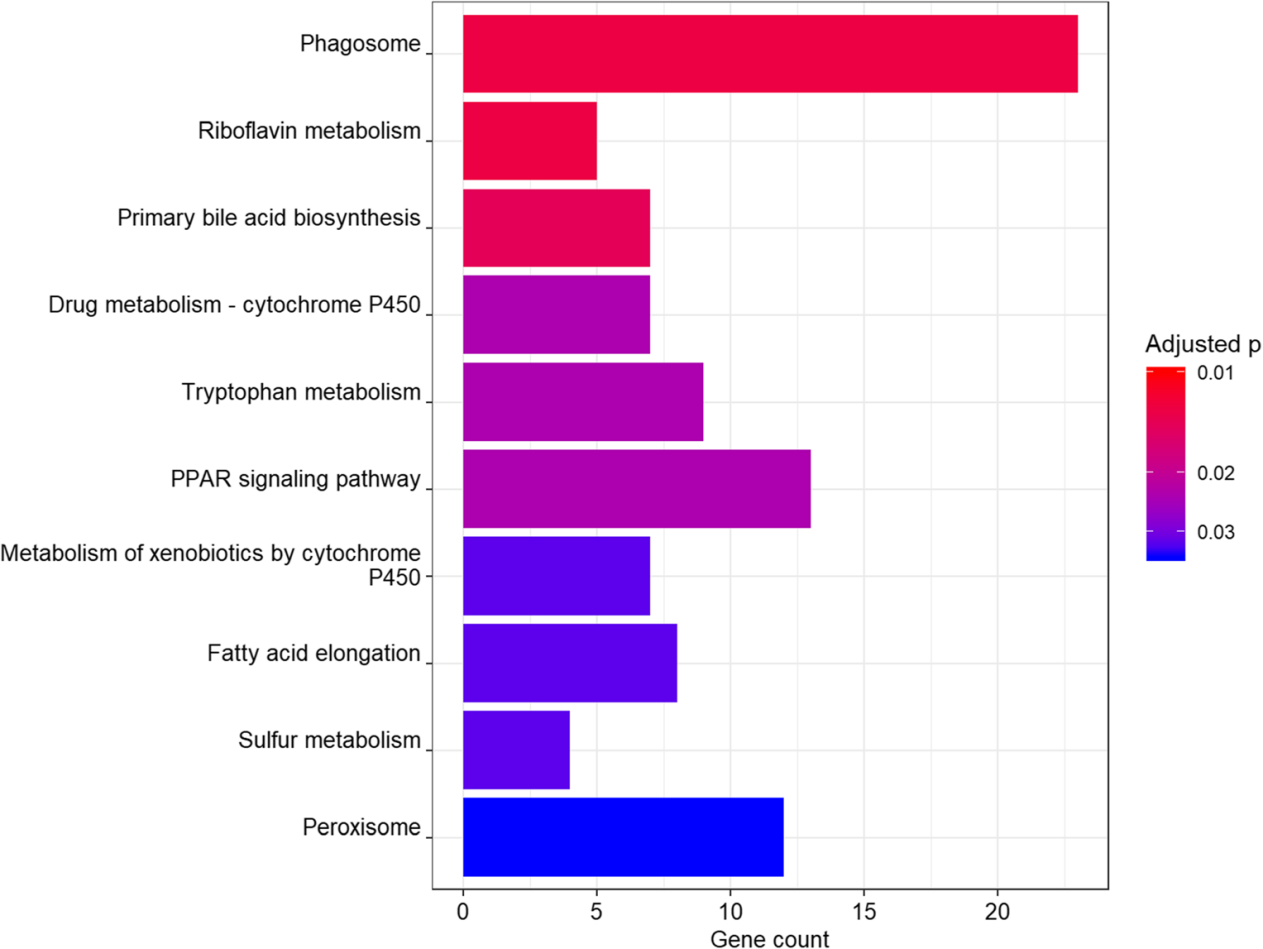
Bar plot of enriched KEGG pathways between pylorus tissue of red and white Chinook salmon. Bars are colored by significance (false discovery adjusted *p* value). X axis is number of DE genes per pathway

**Fig. 8:**
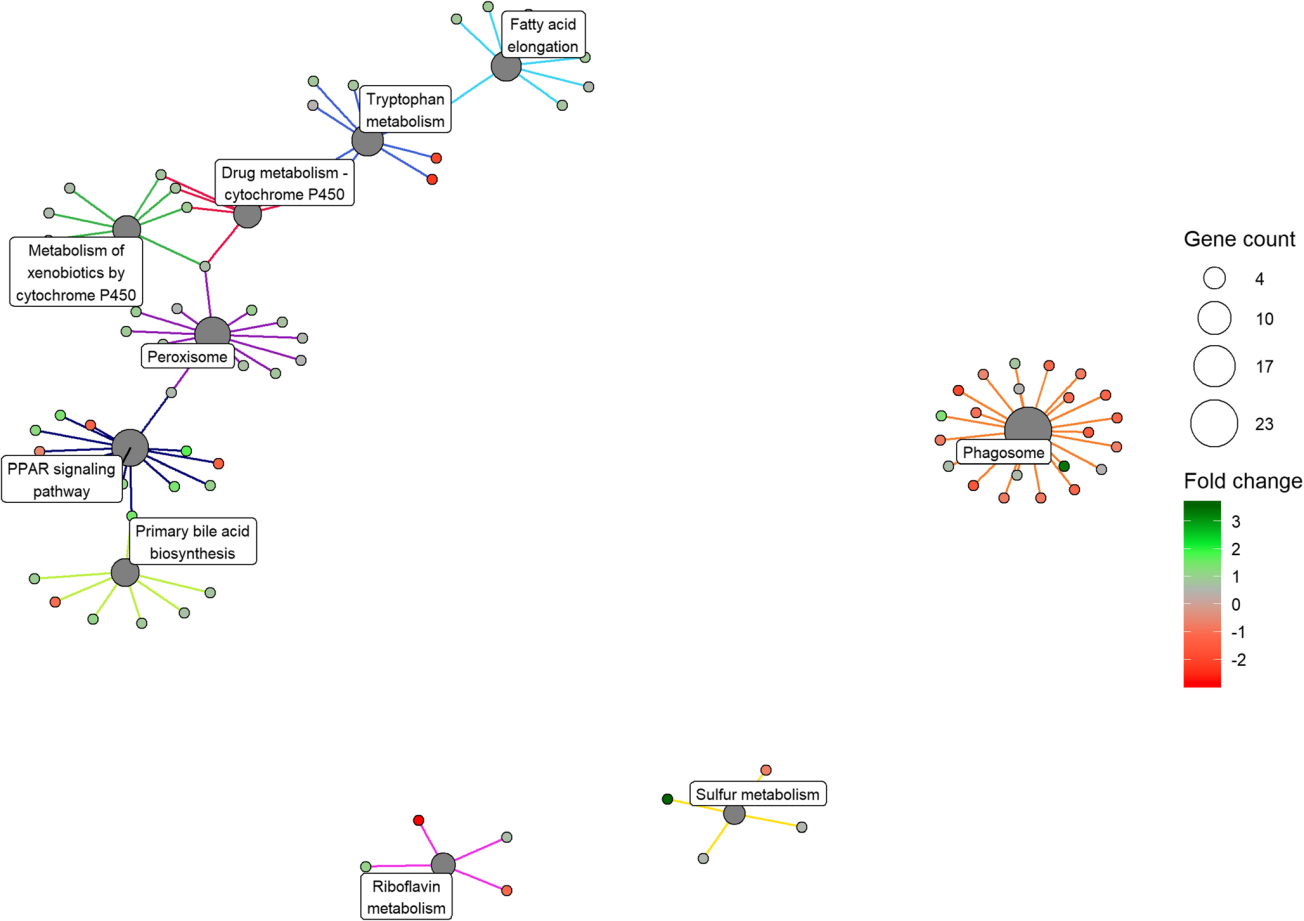
Concept network of enriched KEGG pathways between treatment groups pylorus_white and pylorus_red. DE genes found in each pathway are colored by log fold change and dot size of pathway indicates the number of DE genes in that pathway (green = upregulated, red = downregulated). Links between pathways and their associated DE genes are x-axis represents the log2 fold change of mean read counts per gene between red and white Chinook liver colored by pathway.

KEGG pathways were enriched for differentially expressed genes between red and white pylorus tissue, but not in muscle or liver tissue. In the pylorus, 10 pathways were significantly enriched (Fig. 7): 23 genes in the Phagosome pathway (FDR adjusted *p* = 1.3e-02), 13 genes in the PPAR signaling pathway (adj.*p* = 2.3e-02), 12 genes in the Peroxisome pathway (adj.*p* = 3.6e-02), 9 genes for the Tryptophan metabolism pathway (adj.*p* = 2.3e-02), 7 genes involved in the Metabolism of xenobiotics by cytochrome P450 (adj.*p* = 3.2e-02), 7 involved in the Drug metabolism—cytochrome P450 pathway (adj.*p* = 3.2e-02); 7 in the Primary bile acid biosynthesis pathway (adj.*p* = 1.4e-02), 5 genes for the Riboflavin metabolism pathway (*p* = 1.3e-02), and finally 4 genes involved in the Sulfur metabolism pathway (adj.*p* = 3.2e-02). Table 1 shows each enriched KEGG pathway and Supplementary Table 6 contains a table listing each differentially expressed genes per pathway. Furthermore, several pathways are linked by few associated differentially expressed genes as displayed in the concept network (Fig. 8).

**Table 1:** Enriched KEGG pathway in Chinook pylorus

Pathway ID	Pathway description	adjusted *p*	Gene count
otw04145	Phagosome	1.4e-02	23
otw00740	Riboflavin metabolism	1.4e-02	5
otw00120	Primary bile acid biosynthesis	1.6e-02	7
otw00982	Drug metabolism - cytochrome P450	2.6e-02	7
otw00380	Tryptophan metabolism	2.6e-02	9
otw03320	PPAR signaling pathway	2.7e-02	13
otw00980	Metabolism of xenobiotics by cytochrome P450	3.5e-02	7
otw00920	Sulfur metabolism	3.5e-02	4
otw00062	Fatty acid elongation	3.5e-02	8
otw04146	Peroxisome	4.0e-02	12

## Discussion

The current study directly compared muscle, liver, and pylorus transcriptome profiles of two Chinook salmon phenotypes, red-pigmented and white-unpigmented muscle. Both phenotypes were reared under the same experimental conditions and fed with the same diets supplemented with astaxanthin (100 mg/kg). There were relatively few differentially expressed genes in muscle and liver. On the other hand, in the pylorus, KEEG-enriched pathways showed some noteworthy differences between the two groups. Most regulated apparated to be metabolism and function of the phagosome (Supplementary Material 2), primary bile acid biosynthesis (Supplementary Material 3), cytochrome p450 (Supplementary Material 4 and 5), tryptophan metabolism (Supplementary Material 6), PPAR signaling pathway (Supplementary Material 7), peroxisome function (Supplementary Material 8), sulfur metabolism (Supplementary Material 9), fatty acid elongation (Supplementary Material 10), and riboflavin metabolism (Supplementary Material 11). While several differentially expressed genes expressed in the three tissues are associated with carotenoid absorption, metabolism, and binding, for many others listed (Supplementary Tables 3, 4, and 5), their function is unfortunately yet unknown.

It is possible that the differences in pigmentation between white and red Chinook salmon relies on differences in intestinal astaxanthin absorption, degradation, or improved incorporation into lipoproteins. Carotenoid solubility in lipid is an important physical characteristic, and dietary lipid promotes carotenoid absorption (Torrissen 1985; Torrissen et al. 1990; Choubert et al. 1991). We found no differences in pancreatic digestive enzymes, like bile stimulated lipase, that could have led to variations in the free fatty acids. However, there was a clear differential expression of 7 genes in the lineage for synthetizing bile salts in red chinook intestine. Interestingly, we would expect such enzymes in the liver and not in the pyloric caeca. For instance, in the pyloric caeca, there was a significant differential expression of cholesterol 24-hydroxylase a monooxygenase of the cytochrome P450 family. In mammal’s brain, the role of this enzymes in the cholesterol turnover is well described (Farooqui 2011). Its cholesterol-derived metabolite 24(S)-hydroxycholesterol binds to various targets including the liver X receptors (LXR) and oxysterol-binding proteins. LXR ligands increase cholesterol efflux through expression of apolipoprotein E and D and ATP-binding cassette transporters (reviewd in Farooqui 2011). In addition, hydroxycholesterols and their metabolites play a role in several biological processes, including differentiation, exocytosis, enzyme activities, and immune function (Schroepfer and Wilson 2000). Thus, it is not excluded that these enzymes may have a specific function also in pyloric ceaca. Carotenoids are solubilized into mixed micelles along with other dietary components such as triacylglycerols and their hydrolysis products, phospholipids, cholesterol esters, and bile acid (Deming and Erdman 1999). Bile salts function as micellar solubilizers and may also be required for interaction with the cell membrane or as a transport carrier. It is likely that these enzymes in the red Chinook salmon intestine increase the solubilization of astaxanthin in mixed micelles and thus enhanced astaxanthin uptake. This agrees with our previous findings where diets supplemented with taurocholic acid increased astaxanthin blood levels in Atlantic salmon (Olsen et al. 2005)

The uptake of astaxanthin across the brush border membrane into the enterocyte has received considerable interest in the carotenoid bioavailability research, particularly the different transporters including scavenger receptor class B member (Scarb1, also known as Sr-b1), cluster of differentiation 36 molecule (Cd36), and Npc1-like intracellular cholesterol transporter 1 (Npc1L1) (Baranski et al. 2010; Sundvold et al. 2011) Moreover, recent studies in Atlantic salmon suggested that the upregulation of a newly discovered paralog of *scarb1*, specifically *scarb1*-2 transporter, is associated with enhanced redness in muscle pigmentation and consequently considered as possible quantitative trait loci (QTL) of pigmentation deposition in the muscle. In line with this, in this study, differentially expressed gene analyses showed an upregulation of *scarb1* in red Chinook (0.75 LFC), while white Chinook had higher levels of expression of *npc1l1*-like (− 1.17 LFC) and scavenger receptor class F2 -like (*scarf2*-like – 1.12 LFC). The mechanisms by which these receptors are involved in carotenoid uptake remains elusive, but it is tempting to suggest that Scarb1 may be involved in astaxanthin uptake in red Chinook, while Npc1l1 is not. In humans, NPC1L1 is a sterol transporter in the intestine, involved in cholesterol absorption, but this has not yet been confirmed in teleosts (Altmann et al. 2004). SCARB-F2 and SCARB-F1 are in humans described as transporter of modified lipids as carbamylated LDL (cLdl), acetylated LDL (AcLDL), or oxydated LDL (OxLDL) particles. SR-F2 lacks scavenger receptor activity but preferentially forms heterodimers with SR-F1 suppressing its ligand-binding properties (Zani et al. 2015). Interestingly, if the same function is conserved in fish, it may possibly lead to a reduction of Scarb1-mediated uptake of carotenoid in white Chinook.

In enterocytes, another process regulating availability of astaxanthin is its metabolism through cytoplasmic β-carotene 15, 15′-dioxygenase (Bco1), mitochondrial β, β-carotene-9,10′-dioxygenase (Bco2). Bco2 performs an asymmetric cleavage of astaxanthin and the resulting products can then be reduced further by retinol dehydrogenase to form 1 mol of retinol per astaxanthin (Helgeland et al. 2014). Any increase in these enzymes would therefore be expected to reduce astaxanthin bioavailability (Lehnert et al. 2019). Interestingly, in white Chinook, *bco2* was almost two-fold upregulated compared with the red phenotype. Accordingly, other studies in mammals showed that a deficiency of BCO2 is associated with carotenoids accumulating in the adipose tissues, such as subcutaneous adipose tissue, which caused occurrence of yellow fat in sheep, cow, and chicken (reviewed in Wu et al. 2016). Thus, elevated expression of *bco2* in the current experiment may suggest a role in astaxanthin degradation in white Chinook salmon. However, we also observed that red Chinook upregulates the expression of *bco1* (0.75 LFC). Recent QTL studies in Atlantic salmon linked flesh color to polymorphisms on a region on chromosome 26 harboring *bco1* and *bco1like* genes (Baranski et al. 2010; Zoric 2017). It is possible that the symmetrical cleavage of astaxanthin into two retinal (vitamin A aldehyde) molecules may represent the main pathway in red Chinook salmon for vitamin A production, however, without affecting red fillet pigmentation.

Once translocated into enterocyte cytosol, carotenoids that are not enzymatically metabolized can be destined for transport into lipoproteins, and perhaps also translocated across the apical (exocytosis) or basolateral membrane (Reboul 2019). It is likely that this process is carried out by fatty acid transport protein (FATP) and fatty acid binding proteins (FABP) as they display a broad ligand specificity (Reboul 2013). Red Chinook had a significant upregulation of *fatp* (1.11 LFC) and both intestinal-type (*i-fabp,* 1.42 LFC) and liver-type fatty acid-binding protein (*l-fabp*, 1.74 LFC) in enterocytes possibly indicating improved lipid-mediated astaxanthin transport capacity. In line with the former statement, red Chinook showed increased differential expression of several enzymes involved in lipid metabolism including acyl-CoA-binding protein (*acil-Coa,* 0.95 LFC) and long-chain acyl-CoA synthetase (*acs,* 1.22 LFC), which may regulate activation, esterification, and transport of fatty acid in the plasma (Mashek et al. 2007; Young et al. 2018).

Furthermore, cleaved and non-cleaved astaxanthin and fatty acid are incorporated into lipoproteins or be directly loaded onto *apoA*/HDL particles by specialized ATP-binding cassette (*abc*) transporters and finally transported into the bloodstream. The white Chinook salmon showed an upregulation (− 1.7 LFC) of abc family g1 member 1–like (*abcg1*) transmembrane transporter, which in mammal is known for mediating cholesterol efflux to HDL (Wang et al. 2013; Westerterp et al. 2016).

Red Chinook salmon also seems to upregulate apolipoprotein a-IV (*apo-aIV* 1.7 LFC). ApoA-IV is in many mammals mainly synthesized in the intestine and is an important constituent of chylomicrons and HDL synthetized and excreted from intestines postprandially (Roman-Padilla et al. 2016; Qu et al. 2019). ApoA-IV also shows many other functions including antioxidants and regulating appetite, satiety, and food intake (Wang et al. 2015). It is not unlikely that upregulation therefore stabilizes astaxanthin and increases its plasma transport. In this respect, it is interesting to note that in a recent study of red and white Chinook salmon, it was shown that SNPs aligned to locations near several Apo genes (i.e. *apo-a1*) (Lehnert 2016).

Enriched pathway analyses also showed differences in the expression of genes involved in riboflavin and tryptophan metabolism. Particularly white salmon showed a significant upregulation of acid phosphatase 1 gene (*acp1*, − 2.86 LFC) required to produce reduced riboflavin (Powers 2003). It is well established that riboflavin is the precursor of coenzymes, flavin mononucleotide (FMN), and flavin adenine dinucleotide (FAD) (Udhayabanu et al. 2017). They serve as an electron carrier in a number of oxidation-reduction (redox) reactions involved in energy production and numerous metabolic pathways including β-oxidation, hormone synthesis, vitamins (vitamins A, C, and B12 and pyridoxine, niacin, and folate), and amino acid metabolism (Depeint et al. 2006). Similarly, tryptophan is an essential amino acid, which is required for the synthesis of proteins, serotonin, and melatonin (Hoseini et al. 2019). When converted into NAD, it functions in a wide range of oxidation-reduction reactions and non-redox reactions. There is no direct relation between either riboflavin and tryptophan in carotenoid metabolism, and to date, many important biological functions of this vitamin makes it difficult to identify a specific role in flesh pigmentation processes based on the limited information in this area.

Genes involved in carotenoid catabolism and excretion can also play a role in tissue deposition of astaxanthin in salmon. Enriched KEGGs pathways showed that cytochrome P450 genes were significantly activated mainly in red Chinook. Astaxanthin apparently stimulates liver CYP gene expression in humans (Kistler et al. 2002) and rats (Wolz et al. 1999; Jewell and O’brien 2019), while this occurs in a species-specific manner (Kistler et al. 2002). In cultured human hepatocytes, major cytochrome 450 enzymes, CYP3A4, and CYP2B6 were induced by astaxanthin, but not other CYPs (i.e. CYP1A) (Kistler et al. 2002). In rainbow trout, the cytochrome P450 system was not involved in astaxanthin metabolism (Page and Davies 2002). Therefore, another enzyme system must be present in these species to process these carotenoids. According to Woggon WD (Woggon 2002), β-carotene dioxygenase does not cleave astaxanthin, as supported by the asymmetric products recovered by Wolz et al. (1999). Kistler et al. (2002) suggested that if cleavage occurs at sites other than C9, C9′, the degradation of the polyene chain must be rapid since intermediary products were not recovered. The cytochrome P450 enzymes CYP26A1, CYP26B1, and CYP26C1 carry out the catabolism of retinoic acid to 4-hydroxy-retinoic acid, 4-oxo-retinoic acid, and 18-hydroxy-retinoic acid (White et al. 2000; White et al. 2007). It is possible that this is because the level of vitamin A included in the basal diet was enough to metabolize the dietary carotenoid load delivered, due to the low carotenoid extraction ratio from the liver (Page and Davies 2003). Further investigation in this area would be required.

Recent molecular studies in birds have clearly shown that cytochrome P450 enzymes also affect their coloration. For example, coloration in zebra finch (*Taeniopygia guttata*) (Mundy et al. 2016), red siskins (*Spinus cucullata*), and common canaries (*Serinus canaria*) (Lopes et al. 2016) is controlled by the cytochrome P450 family gene *Cyp2j19*. This enzyme is considered responsible for carotenoid ketolation and thus ketocarotenoid production, i.e. astaxanthin and canthaxanthin, in the pigmented tissues. In this study, *cyp2j19* was detected in gut but not in muscle or liver, being expressed 1.2 LFC more in red than white Chinook salmon. It appears then, that coloration in bird and fish may be regulated differently by CYP or other enzymes. In fact, white Chinook salmon were able to conserve their characteristic unpigmented muscle even when fed astaxanthin supplemented diet. It is possible that the enzymatic hydrolysis of carotenoid into ketocarotenoid may not be the key process regulating pigmentation in red Chinook salmon. It is likely that differences in Chinook red and white muscle phenotypes are linked to differences in absorption, transport, and metabolism for astaxanthin deposition in muscle.

Carotenoids have been linked with several beneficial biological functions in salmonids including enhanced survival, immune function, and antioxidant status. Interestingly, white Chinook phenotype continue to persist in nature also depositing astaxanthin differently from the analogue red phenotype (Lehnert et al. 2018). It is possible that white Chinook salmon have evolved with certain physiological mechanisms to cope with low concentrations of carotenoids in their muscle and other tissues. Lehnert et al. (2016) found that red- and white-pigmented Chinook showed functional genetic differences at two major histocompatibility complex (MHC) genes, particularly with white phenotype being more heterozygous than red individuals at the MHC II-B1 gene resulting in possible advantage for resistance to a wider range of pathogens. In the current study, among the differentially expressed genes linked with the enriched phagosome pathway, white Chinook also upregulated several phagocytosis-promoting receptors and NADPH oxidase–related genes, which may be involved in cell-mediated inflammation mechanism and in defense against infectious agents-antigen presented.

With great surprise, the current transcriptome data did not highlight any possible mechanism in liver or muscle that could explain variation in metabolism and retention of astaxanthin between red and white Chinook. Although a few genes resulted in differentially expressed between the two groups, we could not link them with any possible mechanism associated to carotenoid, i.e. metabolism and retention, and for most of them, their function is yet unknown. On the other hand, some genes highly differentially expressed between groups deserve some attention. For example, *collagen alpha-1* (*XXVIII,* − 5.76LFC) is a gene that was about 6 times more expressed in muscle, liver (− 6.88 LFC), and gut (− 6.7 LFC) tissue of white Chinook salmon. Knowledge on the role of such collagen within extracellular matrices is little to none. However, changes in collagen types can implicate variation in the structural scaffolds in organs and tissues as well as in cellular function through cell–matrix interactions (Birk and Brückner 2011). Similarly, in both liver and pylorus of white Chinook, *beta-crystalline S-1* gene resulted in an upregulation about 8 and 6 times more than in red salmon, respectively. This gene is part of the same *βγ-crystallin* superfamily with a primary function to contribute to the transparency and refractive power of the lens (Wistow 2012). However, other properties may also be important. For example, αβ-crystallin is a functional stress-induced by small heat-shock protein (sHSP) capable of chaperone-like functions, which also have important interactions with other cellular components including cytoskeleton, for example by promoting actin polymerization (Ghosh et al. 2007).

## Conclusions

Understanding which molecular mechanisms rule flesh pigmentation in salmonids fish is a puzzlingly and yet important research field for both salmon’s evolutionary and the economical meaning. Indeed, flesh pigmentation is an important commercial quality trait that can potentially be enhanced by genetic improvement if the main regulators are identified. In the current study, we attempt to compare the gene expression profile of two Chinook salmon phenotypes, naturally red and white pigmented, reared in a common garden experiment. Surprisingly, transcriptome data showed that there were many differentially expressed regulatory pathways in pylorus, which could be linked with the absorption, metabolism, and transport of Ax in the blood circulation. On the other hand, liver and muscle gene expression profiles displayed almost no differences that could explain variation in muscle pigmentation between the two phenotypes. Further study using gene knockout technique could be employed to test the effect of specific genes on the capacity of salmon to metabolize carotenoid and salmon pigmentation.

## Electronic Supplementary Material

ESM 1 (PNG 67 kb)

High resolution image (TIF 55 kb)

ESM 2 (PNG 76 kb)

High resolution image (TIF 62 kb)

ESM 3 (PNG 77 kb)

High resolution image (TIF 61 kb)

ESM 4 (PNG 63 kb)

ESM 5 (PNG 63 kb)

ESM 6 (PNG 108 kb)

ESM 7 (PNG 78 kb)

ESM 8 (PNG 59 kb)

ESM 9 (PNG 49 kb)

ESM 10 (PNG 60 kb)

ESM 11 (PNG 58 kb)

ESM 12 (PNG 30 kb)

ESM 13 (PDF 77 kb)

ESM 14 (XLSX 13 kb)

ESM 15 (XLSX 11 kb)

ESM 16 (XLSX 11 kb)

ESM 17 (XLSX 78 kb)

ESM 18 (XLSX 14 kb)

## References

[CR1] Aas GH, Bjerkeng B, Storebakken T, Ruyter B (1999). Blood appearance, metabolic transformation and plasma transport proteins of 14C-astaxanthin in Atlantic salmon (*Salmo salar L.*). Fish Physiol Biochem.

[CR2] Altmann SW, Davis HR, Zhu L-J, Yao X, Hoos LM, Tetzloff G, Iyer SPN, Maguire M, Golovko A, Zeng M, Wang L, Murgolo N, Graziano MP (2004). Niemann-pick C1 like 1 protein is critical for intestinal cholesterol absorption. Science.

[CR3] Amaya E, Nickell D (2015) Using feed to enhance the color quality of fish and crustaceans. In: Feed and feeding practices in aquaculture. Woodhead Publishing, Sawston, pp 269–298

[CR4] Baranski M (2015) Heritability of fish coloration. In: Evolutionary biology of the Atlantic Salmon. CRC Press, Boca Raton, pp 206–220

[CR5] Baranski M, Moen T, Våge D (2010) Mapping of quantitative trait loci for flesh colour and growth traits in Atlantic salmon (*Salmo salar*). Genet Sel Evol 42 10.1186/1297-9686-42-17 PMC2900243 20525320

[CR6] Benjamini Y, Hochberg Y (1995). Controlling the false discovery rate: a practical and powerful approach to multiple testing. J R Stat Soc Ser B.

[CR7] Birk DE, Brückner P, Mecham R (2011). No title. The extracellular matrix: an overview.

[CR8] Chimsung N, Lall SP, Tantikitti C, Verlhac-Trichet V, Milley JE (2013) Effects of dietary cholesterol on astaxanthin transport in plasma of Atlantic salmon (*Salmo salar*). Comp Biochem Physiol B Biochem Mol Biol 165:73–81 10.1016/j.cbpb.2013.02.007 23458900

[CR9] Choubert G, de la Noüe J, Blanc JM (1991). Apparent digestibility of canthaxanthin in rainbow trout: effect of dietary fat level, antibiotics and number of pyloric caeca. Aquaculture.

[CR10] Deming DM, Erdman JW (1999). Mammalian carotenoid absorption and metabolism. Pure Appl Chem.

[CR11] Depeint F, Bruce WR, Shangari N, Mehta R, O’Brien PJ (2006). Mitochondrial function and toxicity: role of the B vitamin family on mitochondrial energy metabolism. Chem Biol Interact.

[CR12] Farooqui AA (2011). Cholesterol and Hydroxycholesterol in the brain. Lipid mediators and their metabolism in the brain.

[CR13] Ghosh JG, Houck SA, Clark JI (2007). Interactive sequences in the stress protein and molecular chaperone human αB crystallin recognize and modulate the assembly of filaments. Int J Biochem Cell Biol.

[CR14] Helgeland H, Sandve SR, Torgersen JS, Halle MK, Sundvold H, Omholt S, Våge DI (2014). The evolution and functional divergence of the beta-carotene oxygenase gene family in teleost fish-exemplified by Atlantic salmon. Gene.

[CR15] Helgeland H, Sodeland M, Zoric N, Torgersen JS, Grammes F, von Lintig J, Moen T, Kjøglum S, Lien S, Våge DI (2019) Genomic and functional gene studies suggest a key role of beta-carotene oxygenase 1 like (bco1l) gene in salmon flesh color. Sci Rep 9(1):1–12 10.1038/s41598-019-56438-3 PMC6934663 31882713

[CR16] Hoseini SM, Pérez-Jiménez A, Costas B, Azeredo R, Gesto M (2019). Physiological roles of tryptophan in teleosts: current knowledge and perspectives for future studies. Rev Aquac.

[CR17] Jewell C, O’brien NM (2019) Effect of dietary supplementation with carotenoids on xenobiotic metabolizing enzymes in the liver, lung, kidney and small intestine of the rat. Br J Nutr 81:235–242 10434850

[CR18] Kiefer C, Sumser E, Wernet MF, von Lintig J (2002). A class B scavenger receptor mediates the cellular uptake of carotenoids in drosophila. Proc Natl Acad Sci.

[CR19] Kim D, Langmead B, Salzberg SL (2015). HISAT: a fast spliced aligner with low memory requirements. Nat Methods.

[CR20] Kistler A, Liechti H, Pichard L, Wolz E, Oesterhelt G, Hayes A, Maurel P (2002). Metabolism and CYP-inducer properties of astaxanthin in man and primary human hepatocytes. Arch Toxicol.

[CR21] Lehnert S (2016) Why are salmon red? Proximate and ultimate causes of flesh pigmentation in Chinook salmon. Electronic Theses and Dissertations 5909. http://scholar.uwindsor.ca/etd/5909. Google Scholar. Accessed 1 Nov 2019

[CR22] Lehnert SJ, Pitcher TE, Devlin RH, Heath DD (2016). Red and white Chinook salmon: genetic divergence and mate choice. Mol Ecol.

[CR23] Lehnert SJ, Garver KA, Richard J, Devlin RH, Lajoie C, Pitcher TE, Heath DD (2018). Significant differences in maternal carotenoid provisioning and effects on offspring fitness in Chinook salmon colour morphs. J Evol Biol.

[CR24] Lehnert SJ, Christensen KA, Vandersteen WE, Sakhrani D, Pitcher TE, Heath JW, Koop BF, Heath DD, Devlin RH (2019). Carotenoid pigmentation in salmon: variation in expression at BCO2-l locus controls a key fitness trait affecting red coloration. Proc Biol Sci.

[CR25] Liao Y, Smyth GK, Shi W (2014). featureCounts: an efficient general purpose program for assigning sequence reads to genomic features. Bioinformatics.

[CR26] Lim KC, Yusoff FM, Shariff M, Kamarudin MS (2018). Astaxanthin as feed supplement in aquatic animals. Rev Aquac.

[CR27] Lopes RJ, Johnson JD, Toomey MB, Ferreira MS, Araujo PM, Melo-Ferreira J, Andersson L, Hill GE, Corbo JC, Carneiro M (2016). Genetic basis for red coloration in birds. Curr Biol.

[CR28] Love MI, Huber W, Anders S (2014). Moderated estimation of fold change and dispersion for RNA-seq data with DESeq2. Genome Biol.

[CR29] Mashek DG, Li LO, Coleman RA (2007). Long-chain acyl-CoA synthetases and fatty acid channeling. Futur Lipidol.

[CR30] Matthews SJ, Ross NW, Lall SP, Gill TA (2006). Astaxanthin binding protein in Atlantic salmon. Comp Biochem Physiol B Biochem Mol Biol.

[CR31] Mein JR, Dolnikowski GG, Ernst H, Russell RM, Wang XD (2011). Enzymatic formation of apo-carotenoids from the xanthophyll carotenoids lutein, zeaxanthin and β-cryptoxanthin by ferret carotene-9′, 10′-monooxygenase. Arch Biochem Biophys.

[CR32] Mundy NI, Stapley J, Bennison C, Tucker R, Twyman H, Kim K-W, Burke T, Birkhead TR, Andersson S, Slate J (2016). Red carotenoid coloration in the Zebra finch is controlled by a cytochrome P450 gene cluster. Curr Biol.

[CR33] Olsen RE, Kiessling A, Milley JE, Ross NW, Lall SP (2005). Effect of lipid source and bile salts in diet of Atlantic salmon, Salmo salar L., on astaxanthin blood levels. Aquaculture.

[CR34] Olson JA, Hayaishi O (1965). The enzymatic cleavage of beta-carotene into vitamin a by soluble enzymes of rat liver and intestine. Proc Natl Acad Sci U S A.

[CR35] Page G, Davies S (2002). Astaxanthin and canthaxanthin do not induce liver or kidney xenobiotic-metabolizing enzymes in rainbow trout (*Oncorhynchus mykiss Walbaum*). Comp Biochem Physiol Part C Toxicol Pharmacol.

[CR36] Page G, Davies S (2003). Hepatic carotenoid uptake in rainbow trout (Oncorhynchus mykiss) using an isolated organ perfusion model. Aquaculture.

[CR37] Powers HJ (2003). Riboflavin (vitamin B-2) and health. Am J Clin Nutr.

[CR38] Qu J, Ko C-W, Tso P, Bhargava A (2019). Apolipoprotein A-IV: a multifunctional protein involved in protection against atherosclerosis and diabetes. Cells.

[CR39] Rajasingh H, Våge DI, Pavey SA, Omholt SW (2007). Why are salmonids pink?. Can J Fish Aquat Sci.

[CR40] Reboul E (2013) Absorption of vitamin a and carotenoids by the enterocyte: focus on transport proteins. Nutrients 5(9):3563–3581 10.3390/nu5093563 PMC3798921 24036530

[CR41] Reboul E (2019) Mechanisms of carotenoid intestinal absorption: where do we stand? Nutrients 11(4):838 10.3390/nu11040838 PMC6520933 31013870

[CR42] Roman-Padilla J, Rodríguez-Rua A, Claros MG, Hachero-Cruzado I, Manchado M (2016). Genomic characterization and expression analysis of four apolipoprotein A-IV paralogs in Senegalese sole (*Solea senegalensis Kaup*). Comp Biochem Physiol B Biochem Mol Biol.

[CR43] Saha MR, Ross NW, Gill TA, Olsen RE, Lall SP (2005). Development of a method to assess binding of astaxanthin to Atlantic salmon *Salmo salar L.* muscle proteins. Aquac Res.

[CR44] Schiedt K, Vecchi M, Glinz E (1986). Astaxanthin and its metabolites in wild rainbow trout (Salmo gairdneri R.). Comp Biochem Physiol B Comp Biochem.

[CR45] Schiedt K, Vecchi M, Glinz E, Storebakken T (1988). Metabolism of carotenoids in Salmonids. Part 3. Metabolites of astaxanthin and canthaxanthin in the skin of Atlantic salmon (salmo Salar, L.). Helv Chim Acta.

[CR46] Schroepfer GJ, Wilson WK (2000). Oxysterols: modulators of cholesterol metabolism and other processes. Physiol Rev.

[CR47] Sundvold H, Helgeland H, Baranski M, Omholt SW, Våge DI (2011). Characterisation of a novel paralog of scavenger receptor class B member I (SCARB1) in Atlantic salmon (*Salmo salar*). BMC Genet.

[CR48] Torrissen OJ (1985). Pigmentation of salmonids: factors affecting carotenoid deposition in rainbow trout (*Salmo gairdneri*). Aquaculture.

[CR49] Torrissen OJ (1989). Pigmentation of salmonids: interactions of astaxanthin and canthaxanthin on pigment deposition in rainbow trout. Aquaculture.

[CR50] Torrissen OJ, Hardy RW, Shearer KD, Scott TM, Stone FE (1990). Effects of dietary canthaxanthin level and lipid level on apparent digestibility coefficients for canthaxanthin in rainbow trout (*Oncorhynchus mykiss*). Aquaculture.

[CR51] Twyman H, Andersson S, Mundy NI (2018) Evolution of CYP2J19, a gene involved in colour vision and red coloration in birds: positive selection in the face of conservation and pleiotropy. BMC Evol Biol 18 10.1186/s12862-018-1136-y PMC5812113 29439676

[CR52] Udhayabanu T, Manole A, Rajeshwari M, Varalakshmi P, Houlden H, Ashokkumar B (2017). Riboflavin responsive mitochondrial dysfunction in neurodegenerative diseases. J Clin Med.

[CR53] Wang F, Li G, Gu HM, Zhang DW (2013). Characterization of the role of a highly conserved sequence in ATP binding cassette transporter G (ABCG) family in ABCG1 stability, oligomerization, and trafficking. Biochemistry.

[CR54] Wang F, Kohan AB, Lo C-M, Liu M, Howles P, Tso P (2015). Apolipoprotein A-IV: a protein intimately involved in metabolism. J Lipid Res.

[CR55] Westerterp M, Tsuchiya K, Tattersall IW, Fotakis P, Bochem AE, Molusky MM, Ntonga V, Abramowicz S, Parks JS, Welch CL, Kitajewski J, Accili D, Tall AR (2016). Deficiency of ATP-binding cassette transporters A1 and G1 in endothelial cells accelerates atherosclerosis in mice. Arterioscler Thromb Vasc Biol.

[CR56] White JA, Ramshaw H, Taimi M, Stangle W, Zhang A, Everingham S, Creighton S, Tam SP, Jones G, Petkovich M (2000). Identification of the human cytochrome P450, P450RAI-2, which is predominantly expressed in the adult cerebellum and is responsible for all-trans-retinoic acid metabolism. Proc Natl Acad Sci U S A.

[CR57] White RJ, Nie Q, Lander AD, Schilling TF (2007). Complex regulation of cyp26a1 creates a robust retinoic acid gradient in the zebrafish embryo. PLoS Biol.

[CR58] Wistow G (2012). The human crystallin gene families. Hum Genomics.

[CR59] Withler RE (1986). Genetic variation in carotenoid pigment deposition in the red-fleshed and white-fleshed Chinook salmon (*Oncorhynchus tshawytscha*) of Quesnel River, British Columbia. Can J Genet Cytol.

[CR60] Woggon W-D (2002). Oxidative cleavage of carotenoids catalyzed by enzyme models and beta-carotene 15,15′-monooxygenase. Pure Appl Chem.

[CR61] Wolz E, Liechti H, Notter B, Oesterhelt G, Kistler A (1999). Characterization of metabolites of Astaxanthin in primary cultures of rat hepatocytes. Drug Metab Dispos.

[CR62] Wu L, Guo X, Wang W, Medeiros DM, Clarke SL, Lucas EA, Smith BJ, Lin D (2016). Molecular aspects of β, β-carotene-9′, 10′-oxygenase 2 in carotenoid metabolism and diseases. Exp Biol Med.

[CR63] Young PA, Senkal CE, Suchanek AL, Grevengoed TJ, Lin DD, Zhao L, Crunk AE, Klett EL, Füllekrug J, Obeid LM, Coleman RA (2018). Long-chain acyl-CoA synthetase 1 interacts with key proteins that activate and direct fatty acids into niche hepatic pathways. J Biol Chem.

[CR64] Yu G, Wang L-G, Han Y, He Q-Y (2012). clusterProfiler: an R package for comparing biological themes among gene clusters. OMICS.

[CR65] Yu G, Wang L-G, Yan G-R, He Q-Y (2015). DOSE: an R/bioconductor package for disease ontology semantic and enrichment analysis. Bioinformatics.

[CR66] Zani I, Stephen S, Mughal N, Russell D, Homer-Vanniasinkam S, Wheatcroft S, Ponnambalam S (2015). Scavenger receptor structure and function in health and disease. Cells.

[CR67] Zoric N (2017) Characterization of genes and gene products influencing carotenoid metabolism in Atlantic salmon. Electronic Theses and Dissertations. http://hdl.handle.net/11250/2497990. Accessed 1 Nov 2019

