# Supplementary figures and images for: Red and White Chinook Salmon (Oncorhynchus tshawytscha): Differences in the Transcriptome Profile of Muscle, Liver, and Pylorus
Source: Mar Biotechnol (NY). 2020 Jun 26;22(4):581–93. doi: 10.1007/s10126-020-09980-5 (PMC7366597; doi:10.1007/s10126-020-09980-5)

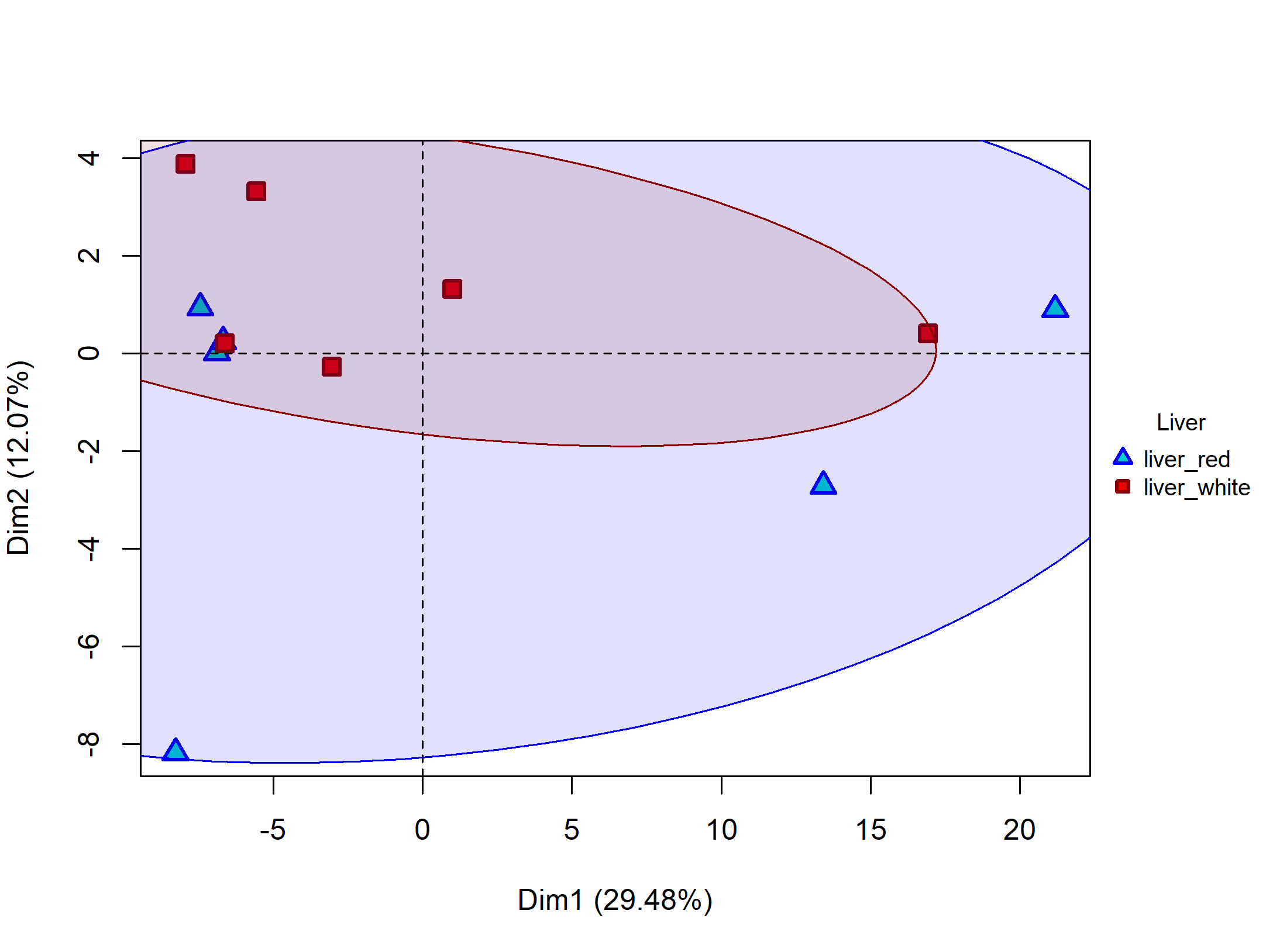

Supplement: Supplementary file 1 — (PNG 67 kb) [file 10126_2020_9980_Fig9_ESM.png]

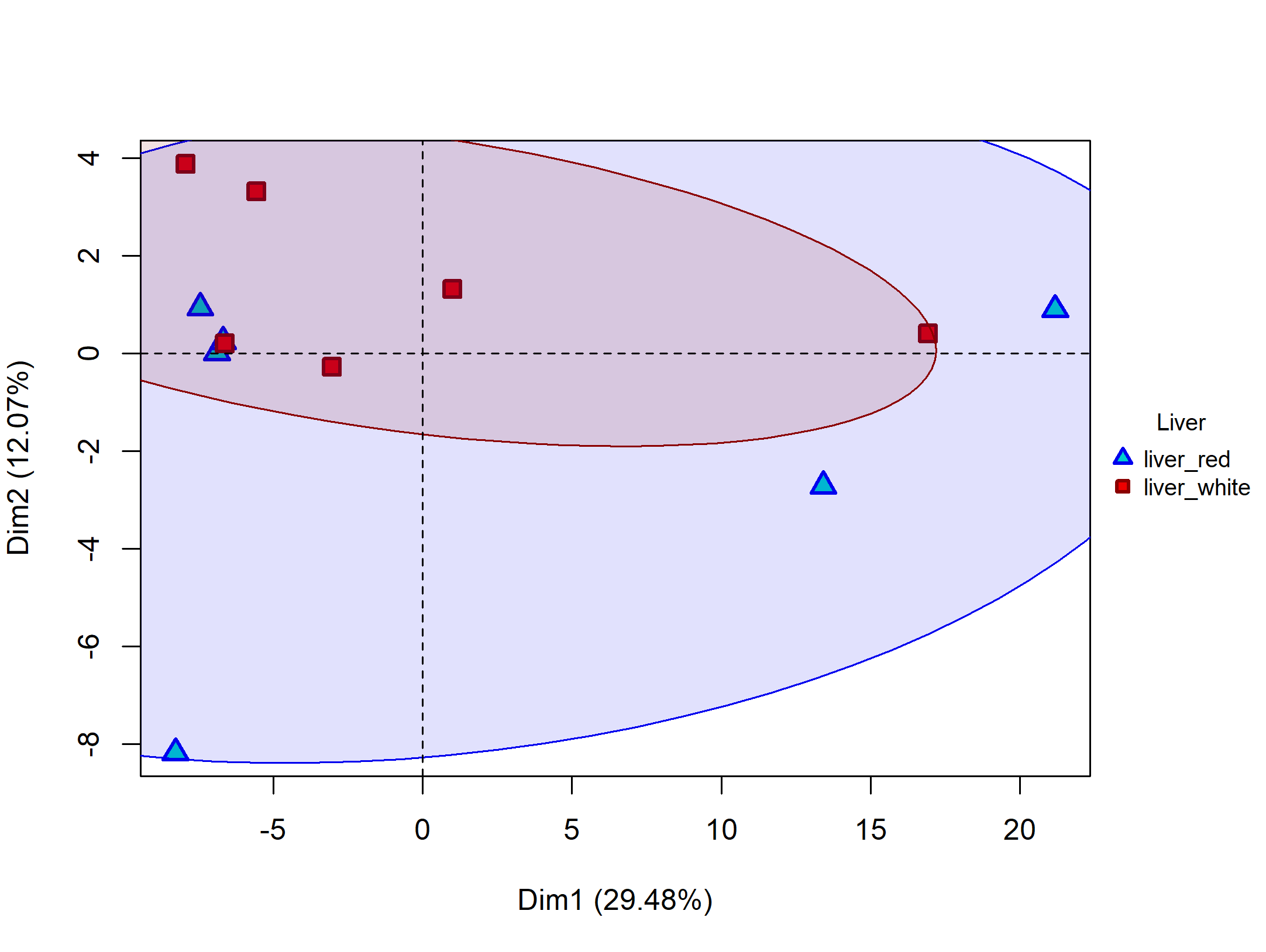

Supplement: Supplementary file 2 — High resolution image (TIF 55 kb) [file 10126_2020_9980_MOESM1_ESM.tif]

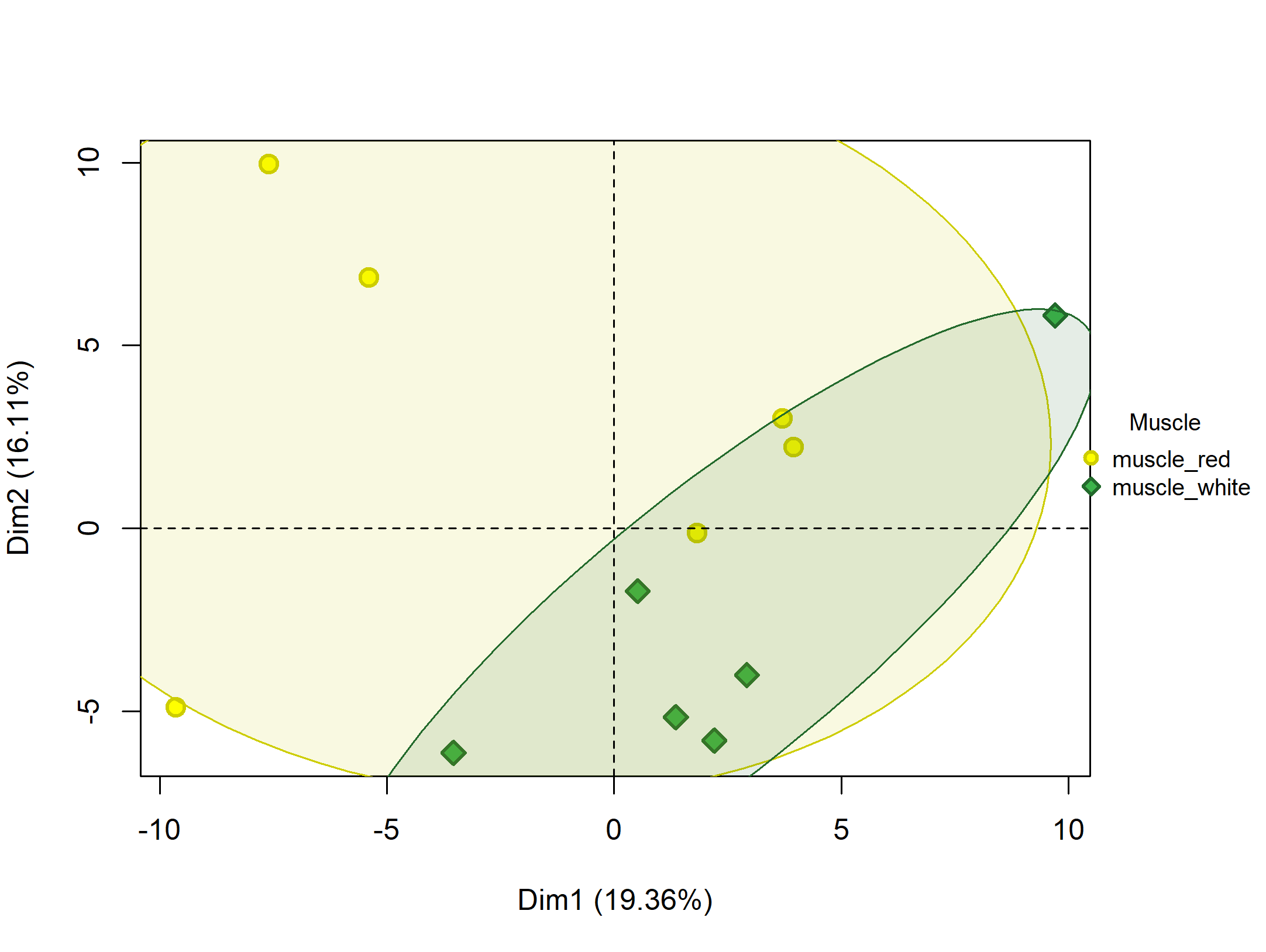

Supplement: Supplementary file 3 — (PNG 76 kb) [file 10126_2020_9980_Fig10_ESM.png]

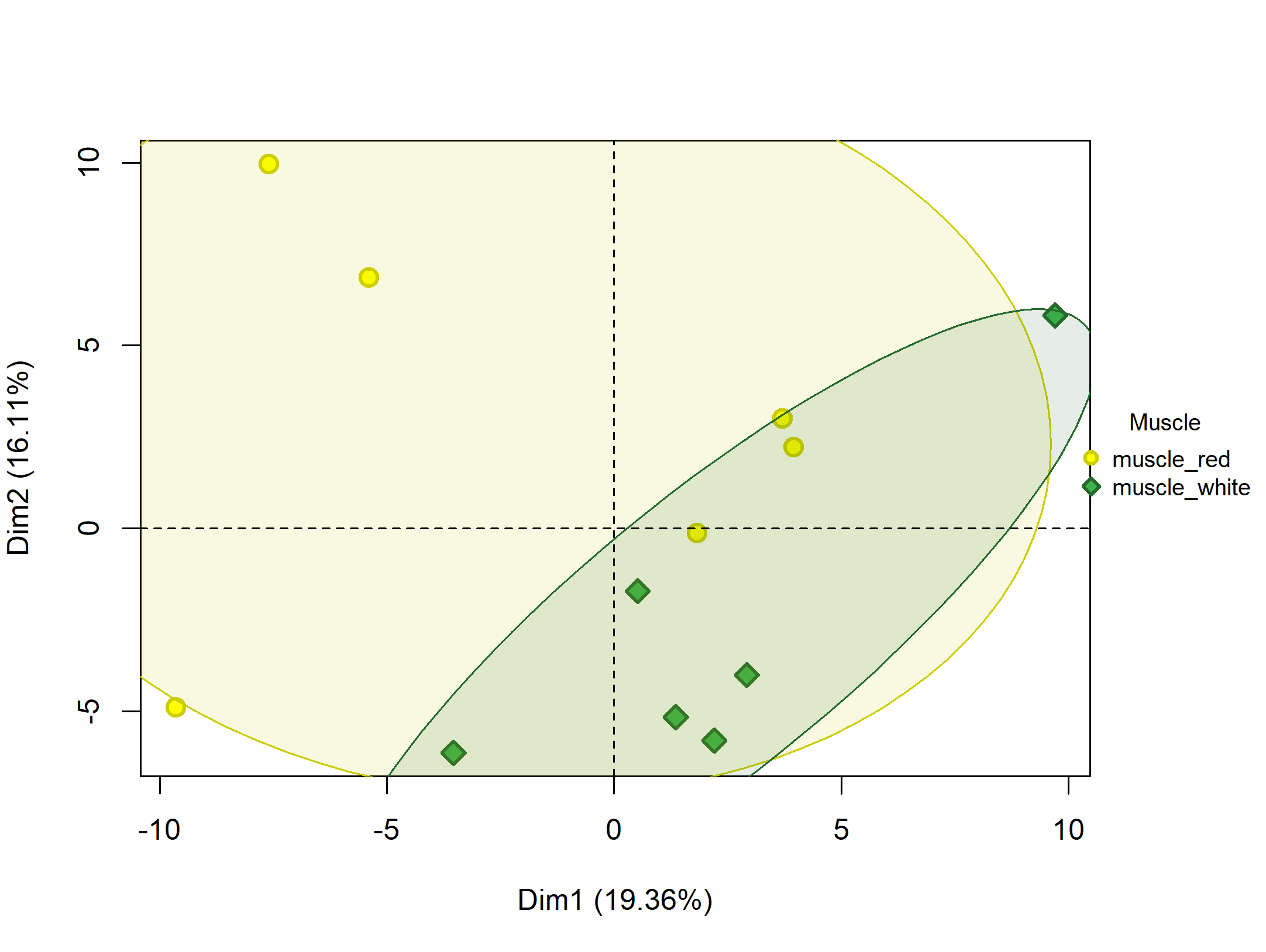

Supplement: Supplementary file 4 — High resolution image (TIF 62 kb) [file 10126_2020_9980_MOESM2_ESM.tif]

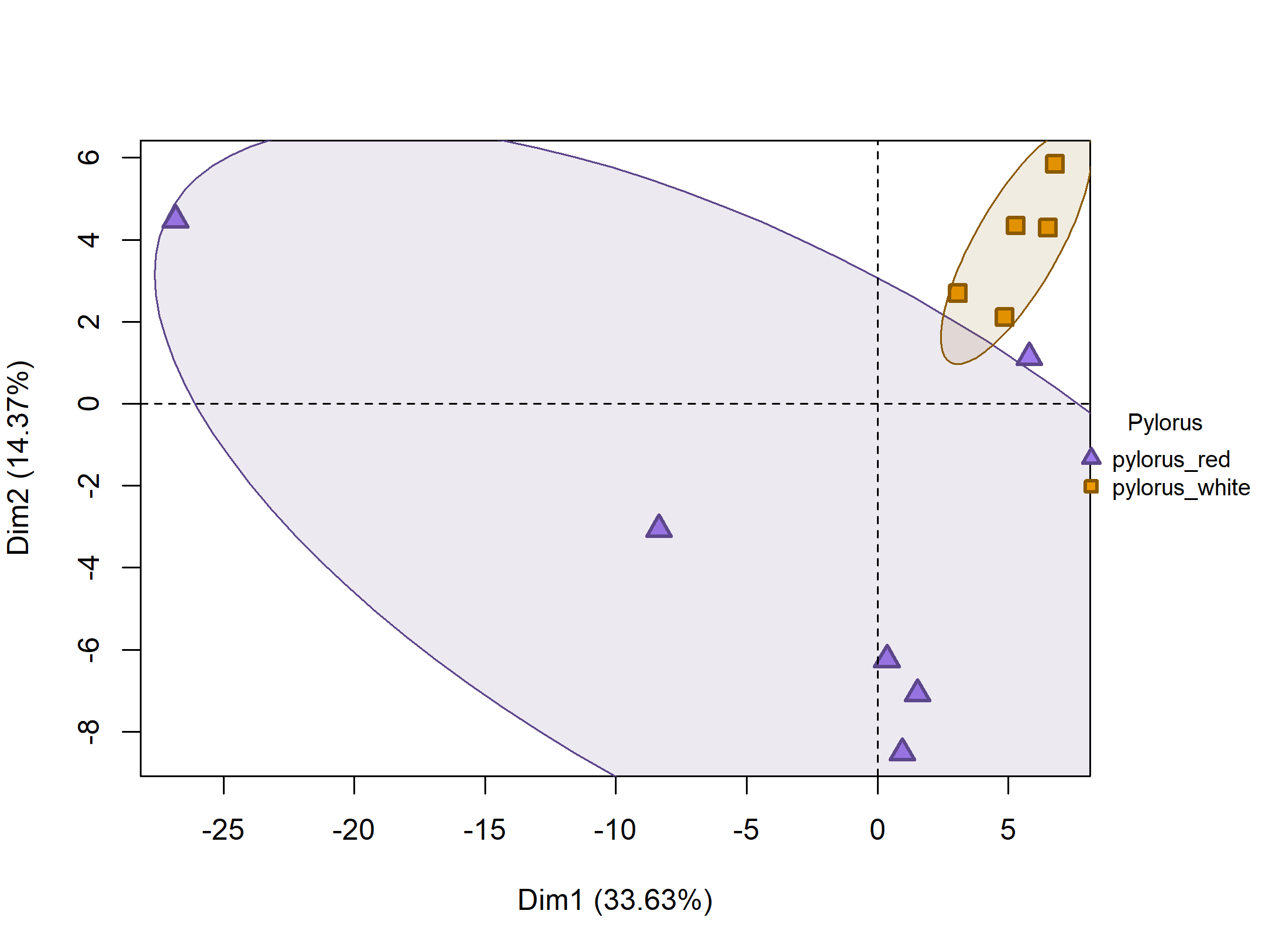

Supplement: Supplementary file 5 — (PNG 77 kb) [file 10126_2020_9980_Fig11_ESM.png]

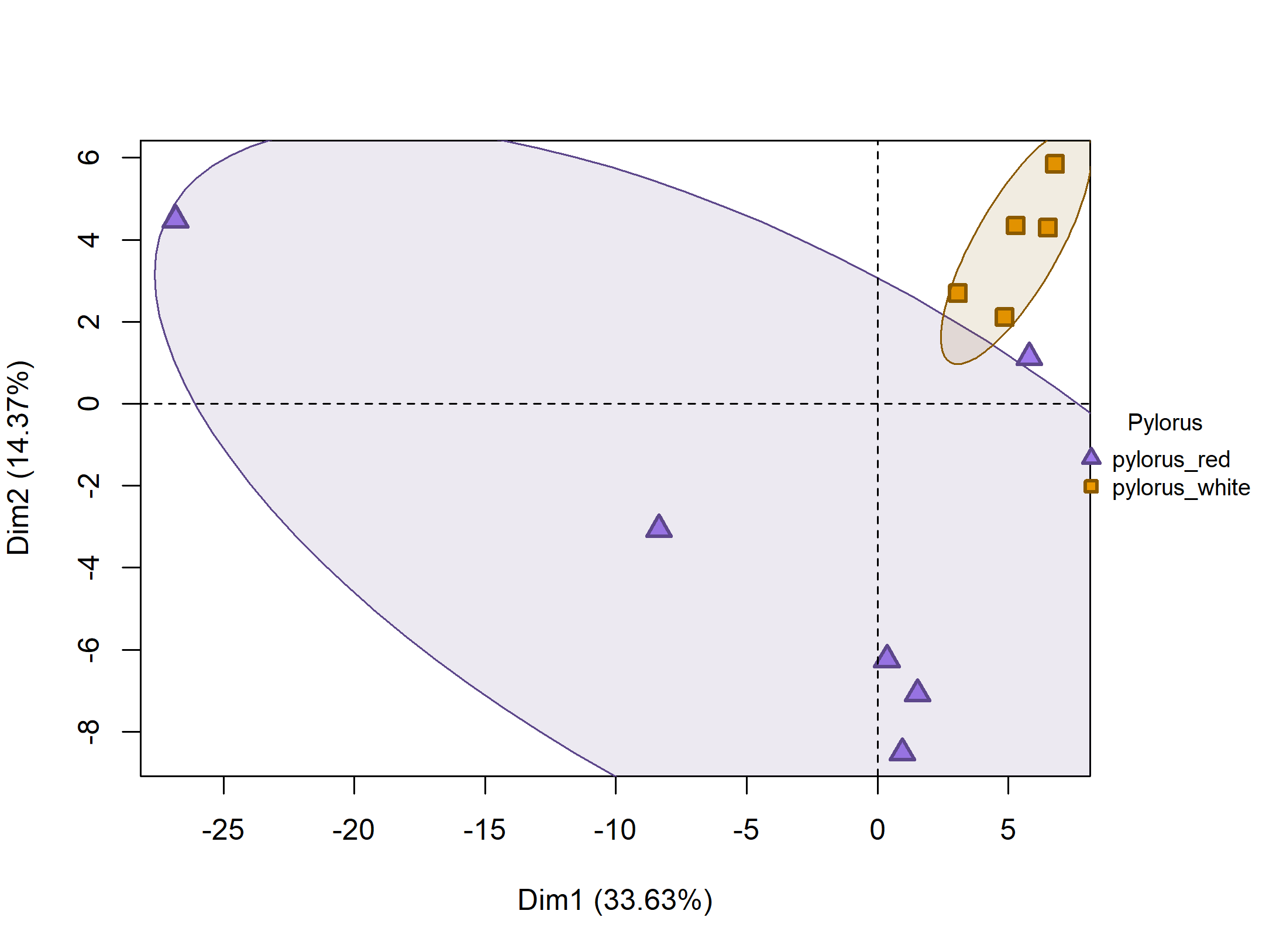

Supplement: Supplementary file 6 — High resolution image (TIF 61 kb) [file 10126_2020_9980_MOESM3_ESM.tif]

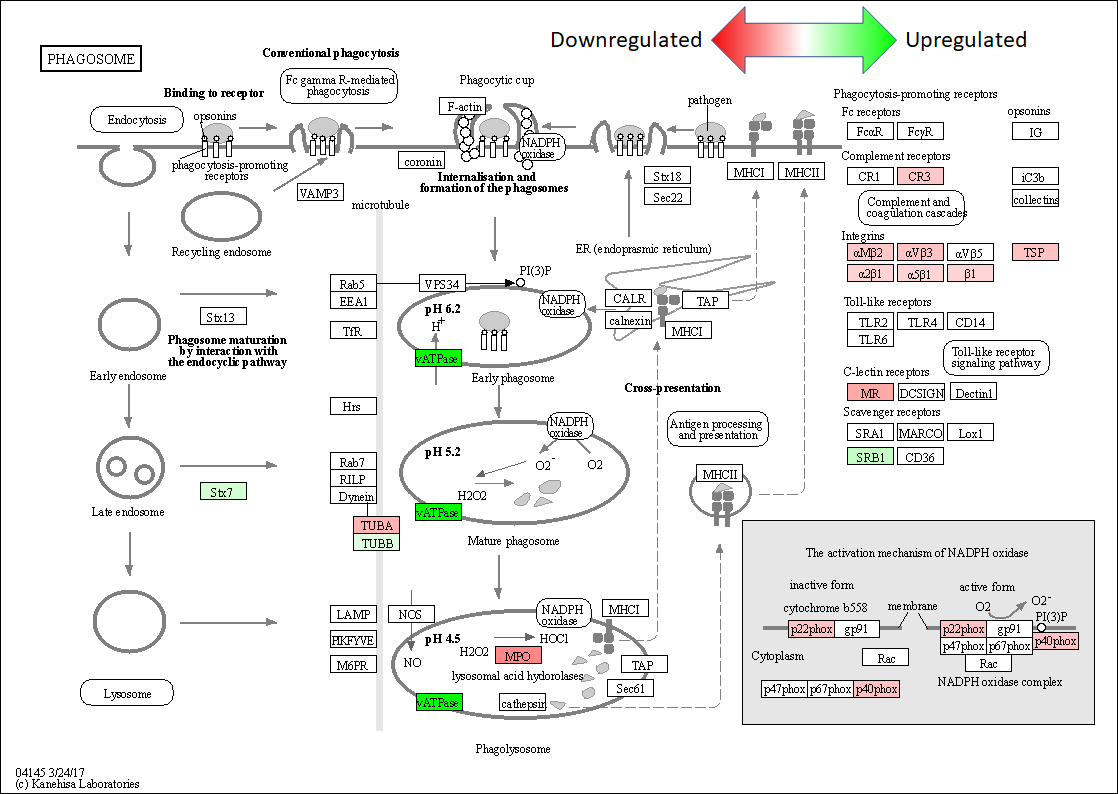

Supplement: Supplementary file 7 — (PNG 63 kb) [file 10126_2020_9980_MOESM4_ESM.png]

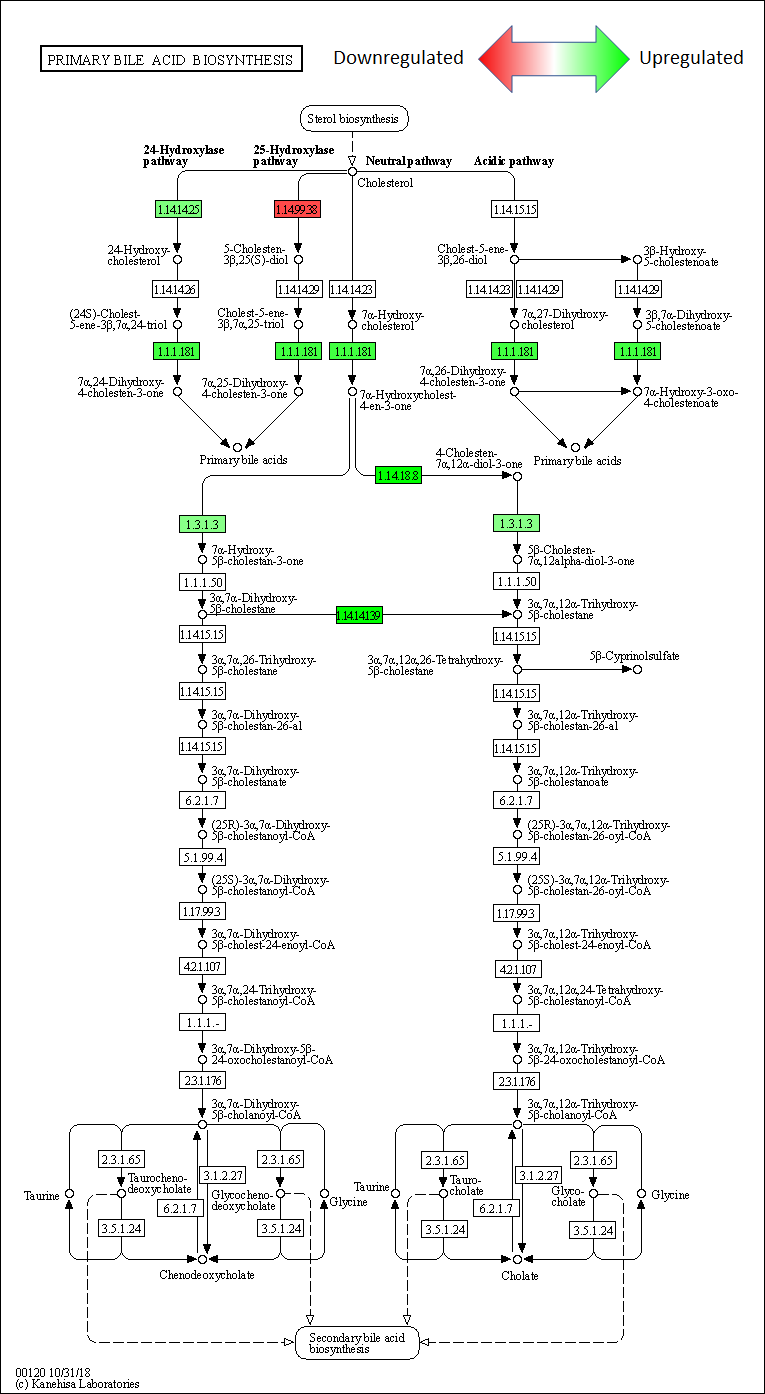

Supplement: Supplementary file 8 — (PNG 63 kb) [file 10126_2020_9980_MOESM5_ESM.png]

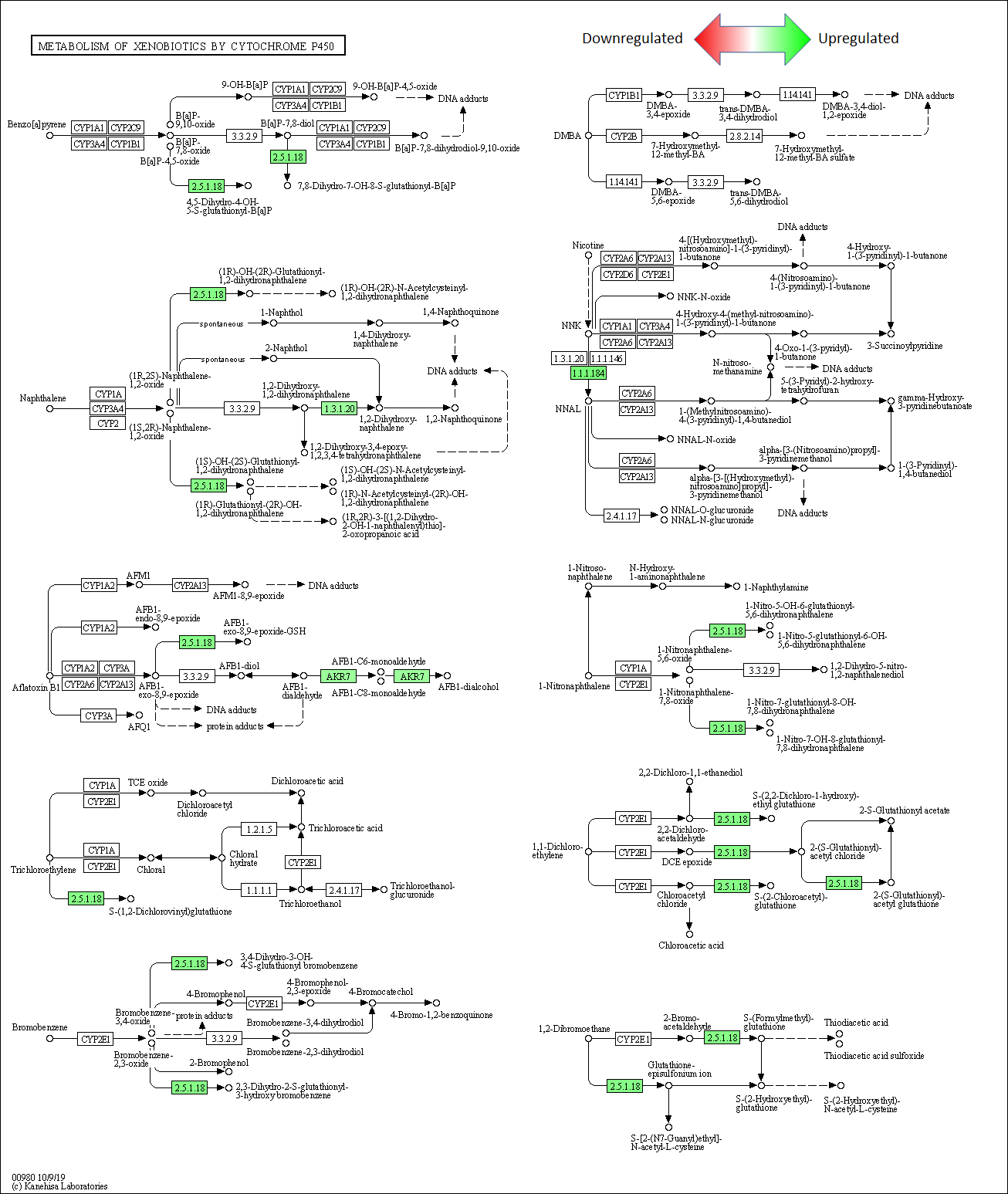

Supplement: Supplementary file 9 — (PNG 108 kb) [file 10126_2020_9980_MOESM6_ESM.png]

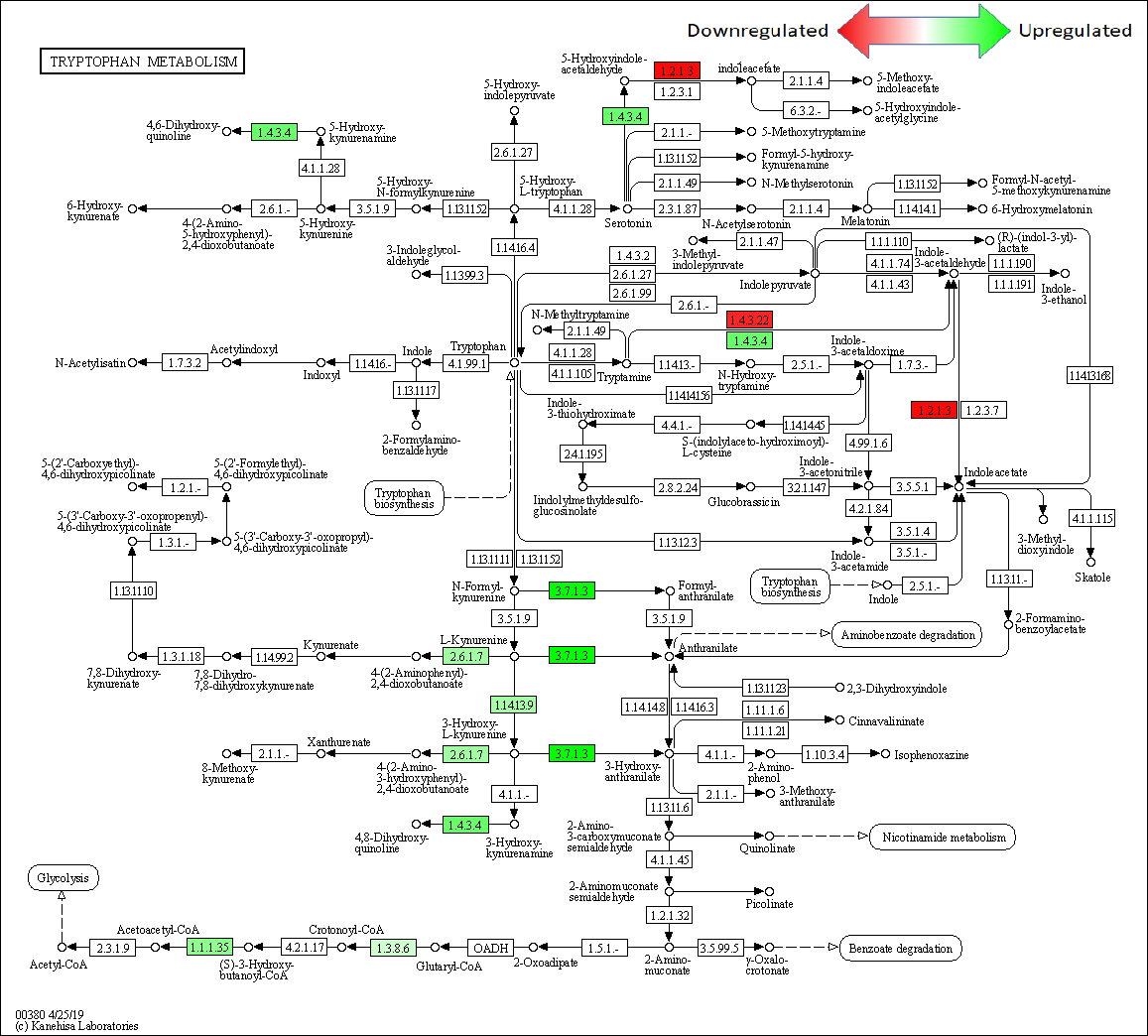

Supplement: Supplementary file 10 — (PNG 78 kb) [file 10126_2020_9980_MOESM7_ESM.png]

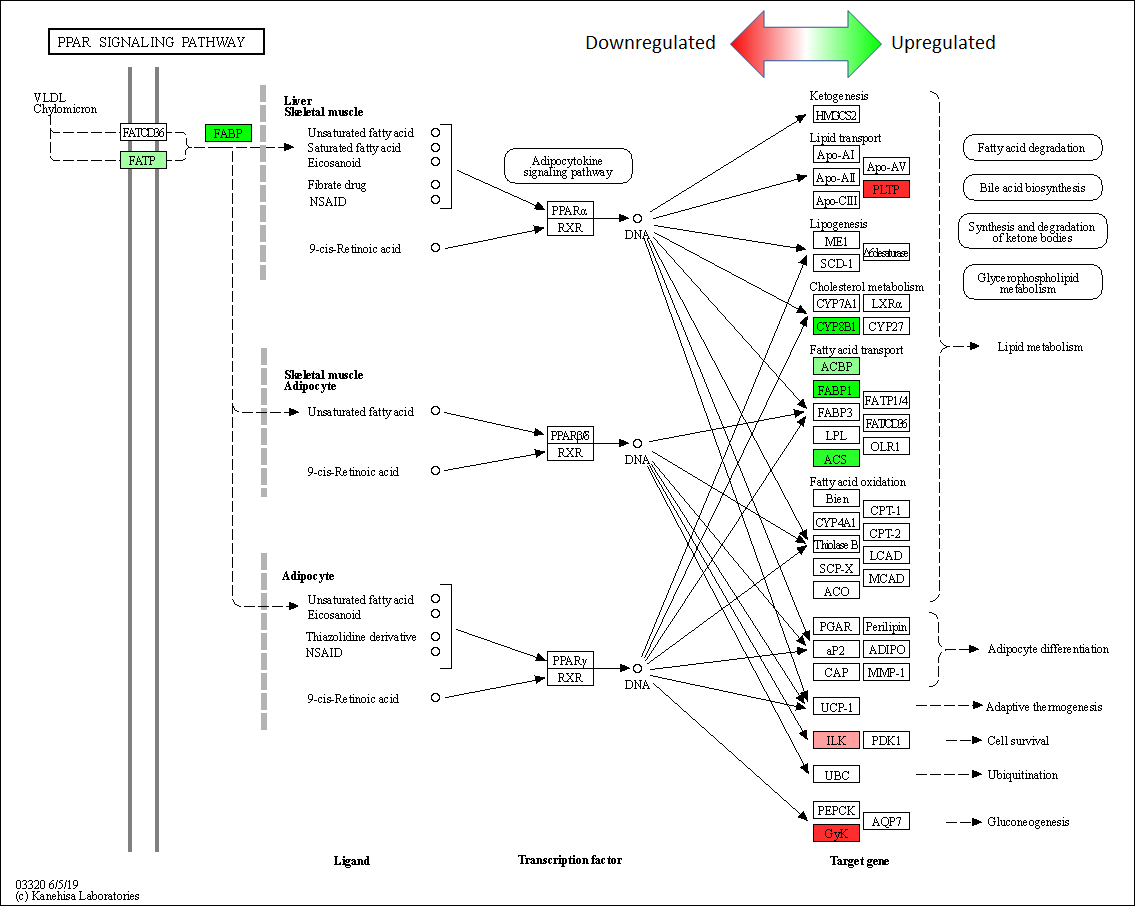

Supplement: Supplementary file 11 — (PNG 59 kb) [file 10126_2020_9980_MOESM8_ESM.png]

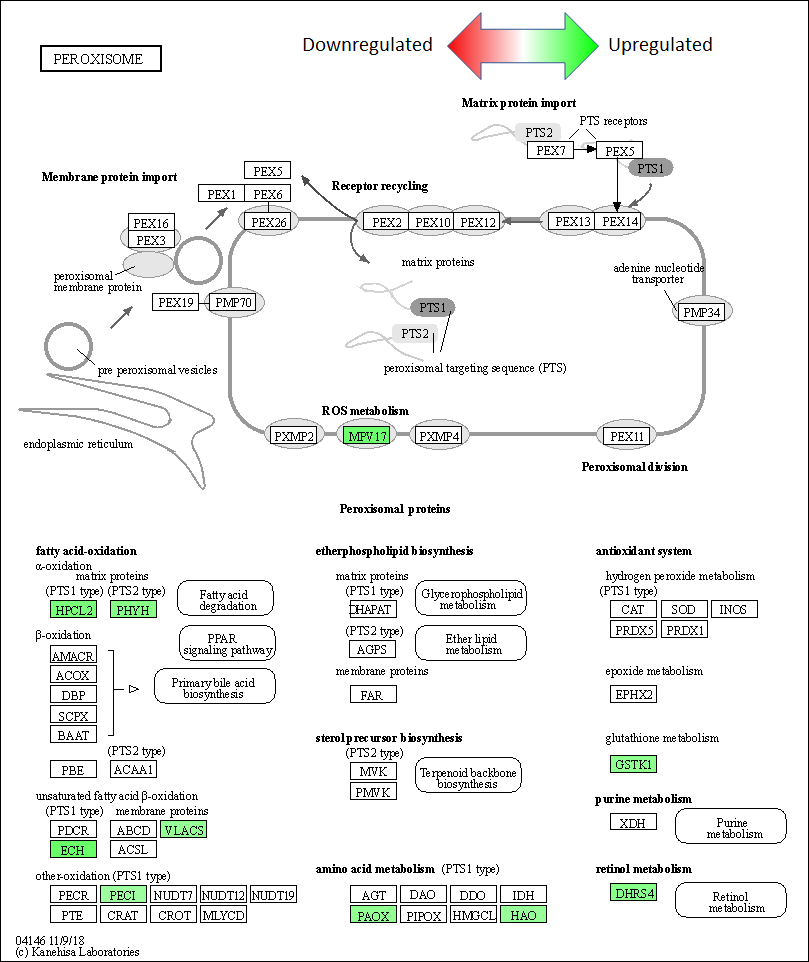

Supplement: Supplementary file 12 — (PNG 49 kb) [file 10126_2020_9980_MOESM9_ESM.png]

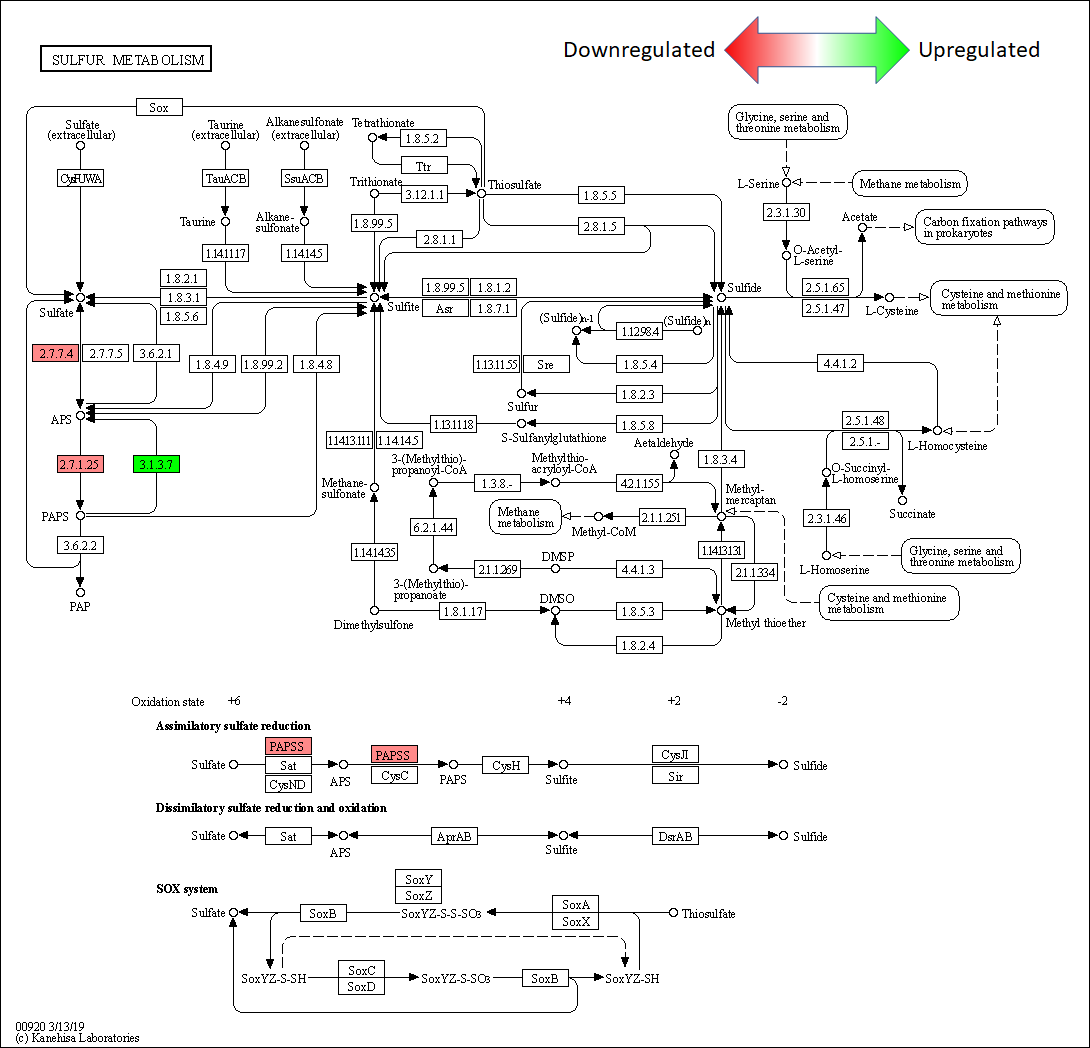

Supplement: Supplementary file 13 — (PNG 60 kb) [file 10126_2020_9980_MOESM10_ESM.png]

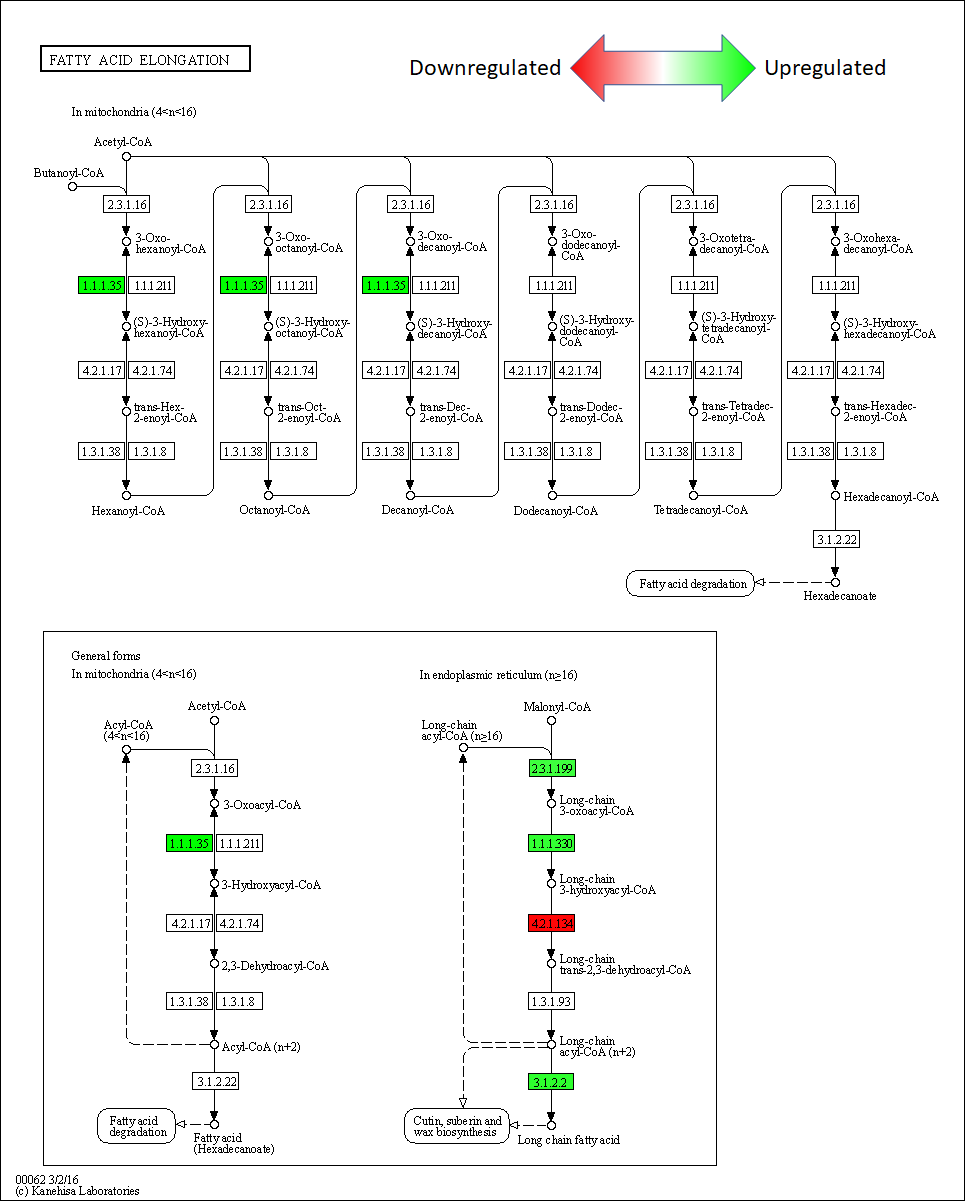

Supplement: Supplementary file 14 — (PNG 58 kb) [file 10126_2020_9980_MOESM11_ESM.png]

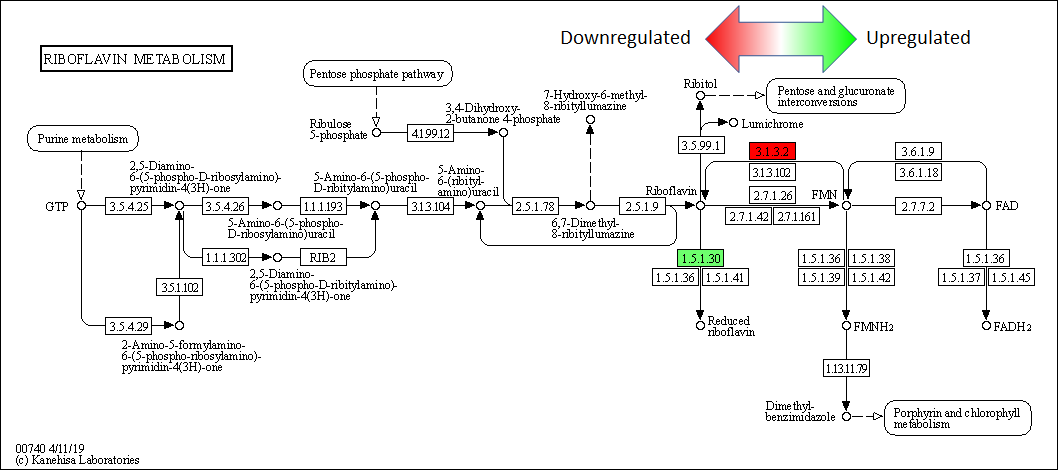

Supplement: Supplementary file 15 — (PNG 30 kb) [file 10126_2020_9980_MOESM12_ESM.png]
