# Supplementary material for: Red and White Chinook Salmon (Oncorhynchus tshawytscha): Differences in the Transcriptome Profile of Muscle, Liver, and Pylorus
Source: Mar Biotechnol (NY). 2020 Jun 26;22(4):581–93. doi: 10.1007/s10126-020-09980-5 (PMC7366597; doi:10.1007/s10126-020-09980-5)
Supplement: Supplementary file 16 — (PDF 77 kb) [file 10126_2020_9980_MOESM13_ESM.pdf]

Exported data

Sample	Total_reads	Chinook_single	Percent	Chinook_multi	Percent	Total_Chinook
C9P	43026841	32230789	74.91%	5854957	13.61%	88.52%
C9M	42429469	27947564	65.87%	9967799	23.49%	89.36%
C9L	43289143	29025950	67.05%	7082443	16.36%	83.41%
C8P	28698634	22146037	77.17%	3694975	12.88%	90.04%
C8M	27752343	18688939	67.34%	6676957	24.06%	91.40%
C8L	32121066	20264776	63.09%	6316885	19.67%	82.75%
C7P	35067752	26276782	74.93%	4404834	12.56%	87.49%
C7M	25828218	17314063	67.04%	6208074	24.04%	91.07%
C7L	32625958	20904225	64.07%	5992911	18.37%	82.44%
C6P	38463945	28175214	73.25%	5137623	13.36%	86.61%
C6M	40764306	27079575	66.43%	9417970	23.10%	89.53%
C6L	38034531	25532573	67.13%	6625155	17.42%	84.55%
C5P	36743890	27353822	74.44%	5180985	14.10%	88.54%
C5M	43069899	28565044	66.32%	1068552	24.81%	91.13%
C5L	38645609	26815893	69.39%	5969709	15.45%	84.84%
C2P	42213200	31551266	74.74%	5720253	13.55%	88.29%
C2M	40865232	27092514	66.30%	1024579	25.07%	91.37%
C2L	39298561	26848647	68.32%	6519810	16.59%	84.91%
C26P	37764160	27489280	72.79%	5513412	14.60%	87.39%
C26M	43653996	28446794	65.16%	1022359	23.42%	88.58%
C26L	41385774	28256331	68.28%	5834673	14.10%	82.37%
C1P	40872224	30982912	75.80%	5484559	13.42%	89.22%
C1M	36836432	25416480	69.00%	8139030	22.10%	91.09%
C1L	39807196	28040821	70.44%	6176977	15.52%	85.96%
C15P	39108931	29049505	74.28%	5351240	13.68%	87.96%
C15M	41142755	26797051	65.13%	1066382	25.92%	91.05%
C15L	43341306	29825394	68.82%	6748233	15.57%	84.39%
C14P	49421659	36852526	74.57%	6602684	13.36%	87.93%
C14M	44061410	29735932	67.49%	9815346	22.28%	89.76%
C14L	45151499	30953105	68.55%	7336682	16.25%	84.80%
C11P	37895675	28187394	74.38%	5569033	14.70%	89.08%
C11M	36066296	23814018	66.03%	8970066	24.87%	90.90%
C11L	36994388	25647288	69.33%	5939650	16.06%	85.38%
C10P	37869101	27917109	73.72%	5158081	13.62%	87.34%
C10M	32996769	22393244	67.86%	7823694	23.71%	91.58%
C10L	38678290	25809259	66.73%	6502664	16.81%	83.54%
